# Chaperone-mediated autophagy plays an important role in regulating retinal progenitor cell homeostasis

**DOI:** 10.1186/s13287-022-02809-z

**Published:** 2022-04-01

**Authors:** Caixia Jin, Qingjian Ou, Jie Chen, Tao Wang, Jieping Zhang, Zhe Wang, Yuanyuan Wang, Haibin Tian, Jing-Ying Xu, Furong Gao, Juan Wang, Jiao Li, Lixia Lu, Guo-Tong Xu

**Affiliations:** 1 Department of Ophthalmology, Shanghai Tenth People’s Hospital, School of Medicine, Tongji University, Shanghai, China; 2 Department of Biochemistry and Molecular Biology, School of Medicine, Tongji University, Shanghai, China; 3 Collaborative Innovation Center for Brain Science, Tongji University, Shanghai, China; 4 Teaching Laboratory Center of Medicine and Life Science, School of Medicine, Tongji University, Shanghai, China

**Keywords:** Retinal progenitor cells, Autophagy, Lysosome, IFITM3, Cell proliferation

## Abstract

**Purpose:**

To explore the function and regulatory mechanism of IFITM3 in mouse neural retinal progenitor cells (mNRPCs), which was found to be very important not only in the development of the retina in embryos but also in NRPCs after birth.

**Methods:**

Published single-cell sequencing data were used to analyze IFITM3 expression in mNRPCs. RNA interference was used to knock down the expression of IFITM3. CCK-8 assays were used to analyze cell viability. RNA-seq was used to assess mRNA expression, as confirmed by real-time quantitative PCR, and immunofluorescence assays and western blots were used to validate the levels of relative proteins, and autophagy flux assay. Lysosomal trackers were used to track the organelle changes.

**Results:**

The results of single-cell sequencing data showed that IFITM3 is highly expressed in the embryo, and after birth, RNA-seq showed high IFITM3 expression in mNRPCs. Proliferation and cell viability were greatly reduced after IFITM3 was knocked down. The cell membrane system and lysosomes were dramatically changed, and lysosomes were activated and evidently agglomerated in RAMP-treated cells. The expression of LAMP1 was significantly increased with lysosome agglomeration after treatment with rapamycin (RAMP). Further detection showed that SQSTM1/P62, HSC70 and LAMP-2A were upregulated, while no significant difference in LC3A/B expression was observed; no autophagic flux was generated.

**Conclusion:**

IFITM3 regulates mNRPC viability and proliferation mainly through chaperone-mediated autophagy (CMA) but not macroautophagy (MA). IFITM3 plays a significant role in maintaining the homeostasis of progenitor cell self-renewal by sustaining low-level activation of CMA to eliminate deleterious factors in cells.

**Supplementary Information:**

The online version contains supplementary material available at 10.1186/s13287-022-02809-z.

## Introduction

Retinal progenitor cells (RPCs) have been investigated for years, and their transplantation or endogenous activation represent promising potential therapeutic avenues in eye disease in the future [1, 2]. Therefore, investigation into the effective amplification of these cells in vitro for a long period [3] and the mechanisms by which they are regulated are particularly important. Here, we analyzed the published dataset of the developing murine retina of single-cell RNA sequencing, and the results suggested that interferon (IFN)-induced transmembrane protein 3 (IFITM3) was highly expressed not only in the development of the retina in embryos but also in the retina after birth [4].

IFITM3 is a transmembrane protein that localizes to several cellular components, including the apical part of the cell, cell surface and endoplasmic reticulum (ER), and is well-known for its role in modulating the interferon-mediated innate immune system to defend against invading pathogenic viruses [5–10]. IFITM3 is involved in germ cell homing and maturation during embryonic development [11–13]. It has been reported that IFITM3 regulates viral infection through autophagy [14, 15]. However, its regulatory mechanism in retinal progenitor cells remains unclear and has not been reported.

Autophagy is involved in regulating the homeostasis of eukaryotic cells [16–20] and plays an important role in maintaining cell proliferation and self-renewal, especially in mesenchymal stem cells (MSCs) [21–23]. Here, we investigated the possible regulatory mechanisms of IFITM3 in mouse neural RPCs (mNRPCs) we established previously [3] through knockdown of IFITM3 expression. The data showed that knockdown of IFITM3 markedly inhibited mNRPC proliferation and cell viability through the chaperone-mediated autophagy (CMA) pathway. When IFITM3 was knocked down, cell proliferation decreased significantly, along with the disruption of the cell membrane transport system and destruction of the cell membrane structure, and the CMA pathway was continuously activated during this process. The results suggested that IFITM3 is a gatekeeper of progenitor cells that keeps the cells healthy during growth and self-renewal. In addition, RNA-seq data suggest that other regulatory factors, such as amino acid metabolism and fat metabolism, were also involved in regulating proliferation after IFITM3 knockdown, which suggests that inhibition of the expression of IFITM3 caused destruction of the cell membrane and eventually induced a metabolic crisis resulting in cell death. Taken together, these data indicate that IFITM3 is the first line of protection for RPC homeostasis and regulates mNRPC viability and proliferation mainly through the CMA pathway.

## Materials and methods

### Cell culture and RNA interference

Adult mouse neural RPCs (mNRPCs) established by our laboratory as our previous description [3] were plated onto dishes coated with 2% Matrigel (Corning, USA) and cultured in 1 × N2/B27 (Thermo Fisher Scientific, USA), 10 ng/ml bFGF (PeproTech, USA), 2 μm CHIR99021 (Selleck Chemicals, USA) and 0.11 mM 2-Mercaptoethanol (Thermo Fisher Scientific, USA) in DMEM/F12 medium (all other materials were from Thermo Fisher Scientific-Gibco, USA). To knock down IFITM3, commercially validated IFITM3 siRNA was synthesized by Proteintech (Rosemont, IL, USA) and transfected into cells using Lipofectamine 3000 (Thermo Fisher Scientific, USA) according to the manufacturer’s protocol. After transfection for approximately 48 h, the cells were collected for further assays. The targeted sequences for the experiments are listed in Additional file 6: Table S1.

### Cell viability assay

For the cell viability assay, cells were seeded at a density of 1 × 10^5^ cells per 100 μL in each well in 96-well microtiter plates (Corning, USA) and transfected with siRNA for 48 h. Cells were grown in each medium in triplicate. Then, 10 μL of reagent from the Cell Counting Kit-8 (CCK-8, TargetMol, USA) was added to each well, and the plates were incubated for 3 h at 37 °C. Cell viability was measured as the absorbance at 450 nm with a microplate reader (iMark™ Microplate Absorbance Reader, BioRad, USA). The mean optical density (OD) values from triplicate wells containing each culture medium were used as the cell viability indices.

### Real-time quantitative PCR

To obtain total RNA, cells were lysed with TRIzol reagent and treated with RNase-free DNase I (both from TaKaRa, China). Reverse transcription (RT) was performed according to our previously published article [3]. Then, quantitative RT-RCR was performed using the SYBR Green Master Mix system (Tiangen Biotech, China). Each PCR mixture contained 10 μL of 2 × SYBR Green Master Mix, sense and antisense primers (Sangon Biotech, China) at 5 μmol/μL, 10 ng of cDNA in a total volume of 20 µl and was subjected to the following protocol: 45 cycles of 95 °C for 15 s, 60 °C for 30 s and 72 °C for 30 s. For relative quantification, 2^−ΔΔCt^ values were calculated and used as an indication of relative expression levels. The IFITM3 gene primer sequences used for RT-PCR were 5′-TGTCCAAACCTTCTTCTCTCC-3′ and 5′-CGTCGCCAACCATCTTCC-3′. The internal control primer sequences for β-actin were 5′-GTGGACATCCGCAAAGAC-3′ and 5′-AAAGGGTGTAACGCAACTA-3′.

### Immunofluorescence assay

Cells were fixed in 4% paraformaldehyde (PFA, Sangon Biotech, China) following the procedure described in our previously published article [3]. At the end of the experiment, the cells were stained with compatible Alexa 488- or Alexa 555-conjugated secondary antibodies (Thermo Fisher Scientific, USA) for 30 min at room temperature. The antibodies are listed in Additional file 6: Table S2.

### Western blot analysis

Cells were harvested and homogenized in RIPA buffer (Beyotime, China) supplemented with protease and phosphatase inhibitor cocktails (TargetMol, USA) on ice and subjected to a previously described protocol [3]. The signal intensities of bands obtained were analyzed by ImageJ (NIH, USA). The antibodies used are listed in Additional file 6: Table S2.

### Bioinformatics analysis

For the single-cell sequence analysis, all cells in the website (https://github.com/gofflab/developing_mouse_retina_scRNASeq) were clustered and visualized using the method of uniform manifold approximation and projection (UMAP) with Seurat (v4.0.4)∷RunUMAP [4, 24]. The annotations of all cells were provided in the dataset. The expression of IFITM3 gene in each sample were visualized using the function FeaturePlot in R package Seurat (v4.0.4).

For the RNA sequence analysis, RNA was extracted from mNRPCs and IFITM3-knockdown cells at 48 h with TRIzol reagent (TaKaRa, China). The RNA-seq library was sequenced with an Illumina NovaSeq 6000 PE150. The standard parameters |logFC|> 1 and *P* < 0.05 were used to screen differentially expressed genes (DEGs). The R package clusterProfiler was used for Gene Ontology (GO) and Kyoto Encyclopedia of Genes and Genomes (KEGG) analyses. The results were visualized with GOplot.

### Organelles involved in IFITM3-knockdown cells

After 48 h of transfection with IFITM3 siRNA, cells were treated with or without 100-nM rapamycin (RAMP, Selleck, USA) for 4 h then analyzed after incubation with 50-nM LysoTracker (Beyotime, China), 50-nM MitoTracker (Beyotime) and 1-μM ER-Tracker (Beyotime) for 30 min. The nuclei were stained with Hoechst 33342 (Sigma-Aldrich, Germany).

### LC3-GFP-mCherry construction and infection and autophagy flux assay

The Ad-mCherry-GFP-LC3 plasmid was purchased from Addgene (plasmid #110060), amplified and sequenced following instructions from Addgene using the following sequencing primers: forward: 5′-CGCGGATCCGGTCGCCACCATGGTGAGCAAGGGCGAG-3′, reverse: 5′-CGCGGCGCGCCGCTGGGTCTAGATGCATGC-3′. The PCR fragment was sequenced to verify that no errors had been introduced. LC3-GFP-mCherry lentivirus was produced by transfection in 293 T cells. The 293 T cells were cultured in DMEM/F12 medium supplemented with 10% fetal bovine serum (FBS, ExCell Bio, China). The virus-containing supernatant was collected after 48 h of transfection and subjected to a previously described protocol [3]. GFP and mCherry expression was verified by microscopy. The cells were treated with 100-nM RAMP to study autophagic flux after IFITM3 knockdown for 48 h. Then, the expression of GFP and mCherry was observed after staining with Hoechst 33342 (Sigma-Aldrich, Germany).

### Statistical analysis

All data are expressed as the mean ± SEM. All analyses were performed with GraphPad Prism 9.3 software. One-way ANOVA was employed for the statistical comparisons. A value of *P* < 0.05 was considered to indicate statistical significance.

## Results

### IFITM3 is involved in mNRPC proliferation

**Fig. 1:**
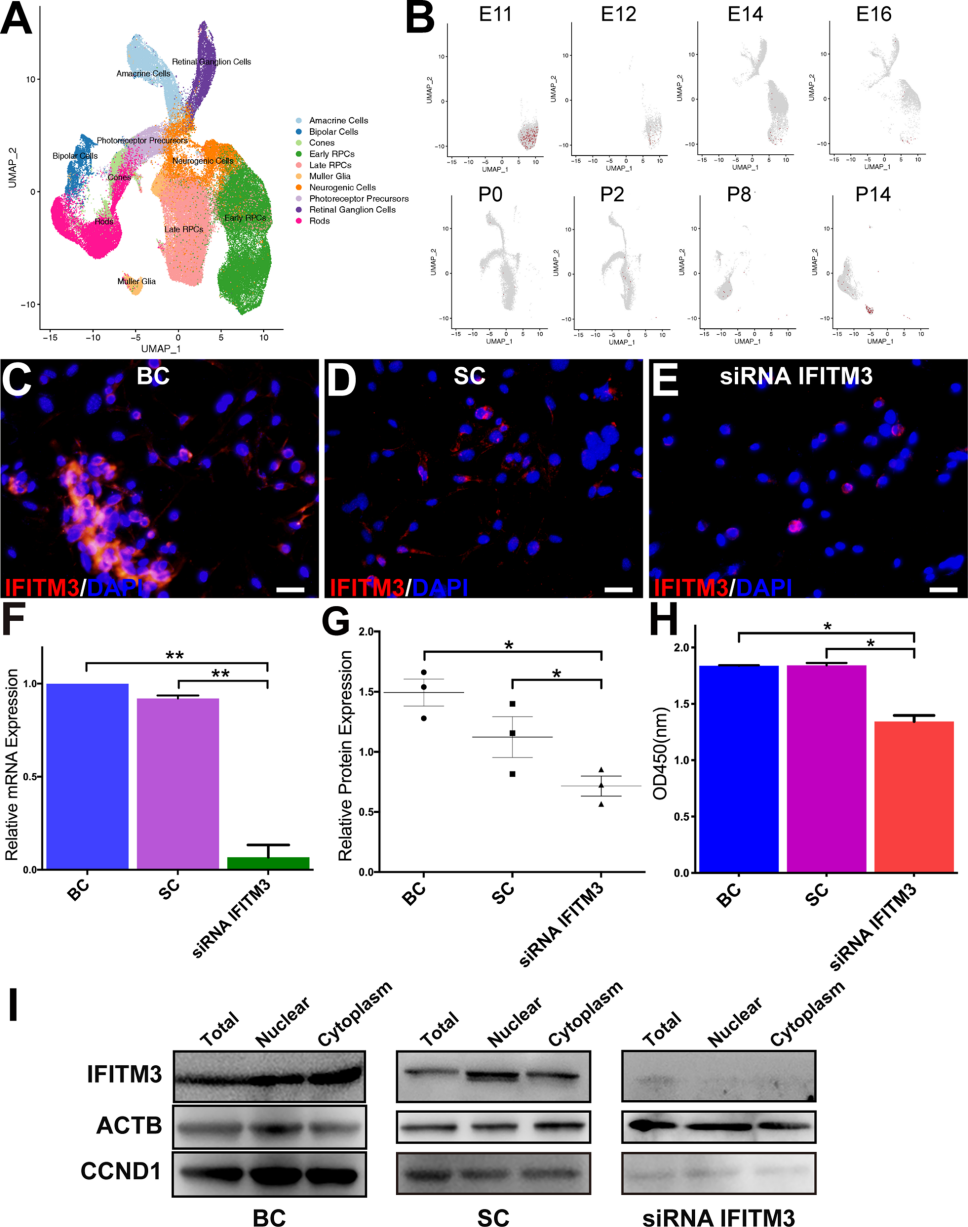
mNRPC proliferation was greatly inhibited after IFITM3 knockdown for 48 h. **A** DimPlot showing the cell types in the murine retina during the embryonic stage and postnatal stage. **B** FeaturePlot showing the expression and distribution of IFITM3 at E11, E12, E14, E16, P0, P2, P8 and P14. **C**–**E** Immunofluorescence assays showed decreased expression of IFITM3 in mNRPCs. Magnification: × 400; scale bar: 50 μm. **F** qRT–PCR showed decreased expression of IFITM3 in mNRPCs. **G** The results of WB assays were consistent with the qPCR results. **H** The CCK-8 assay was used to assess cells after IFITM3 knockdown for 48 h. Data are presented as the mean ± SD (*n* = 3). *P < 0.05; **P < 0.01 (one-way ANOVA and Sidak’s multiple comparisons test). **I** Cell division-related protein expression was decreased in IFITM3-knockdown cells, as determined by WB assays. The results are representative of at least three independent experiments, and representative blots are shown. Abbreviation: BC, blank control; SC, scramble control

Initially, to obtain information on the variation and distribution of the IFITM3 gene during the development of the mouse retina in vivo, we integrated a published dataset of the developing murine retina in single-cell RNA sequencing and visualized the cell-type identification in Fig. 1A [4]. During the embryonic stage, the IFITM3 gene was mainly expressed in the early RPCs at embryological days 11 (E11) and E12 (Fig. 1B). For the postnatal stage (P0, P2, P8), fewer positive expressed in the detected cells, but it also expressed in RPCs and Müller cells 14 days after birth. These results suggest that IFITM3 is important for retinal progenitor cells in retinal development.

To investigate the function of IFITM3 in mNRPCs, we transfected siRNA IFITM3 into the cells and examined cell viability. Cell growth (Fig. 1C–E; Additional file 1: Figure S1A-S1C) was clearly inhibited in the IFITM3 siRNA-transfected group (siRNA IFITM3) compared with the c group and scramble control (SC) group, with significantly decreased expression of IFITM3 at the RNA (Fig. 1F) and protein levels (Fig. 1G) after IFITM3 knockdown, but there was no significant decrease in ARPE19 cell growth (Additional file 1: Figure S1D-E). The cell viability was greatly decreased after IFITM3 knockdown, as shown by the CCK-8 assay, which significantly differed among the groups (Fig. 1H), and the protein expression of Cyclin D1 (CCND1) decreased in the corresponding cell groups (Fig. 1I). These results indicated that IFITM3 was not only involved in the regulation of cell division and activity of retinal progenitor cells during retinal development but also involved in the proliferation and division of in vitro cultured RPCs*.*

### Cell membrane structure and function were dramatically changed after IFITM3 knockdown

**Fig. 2:**
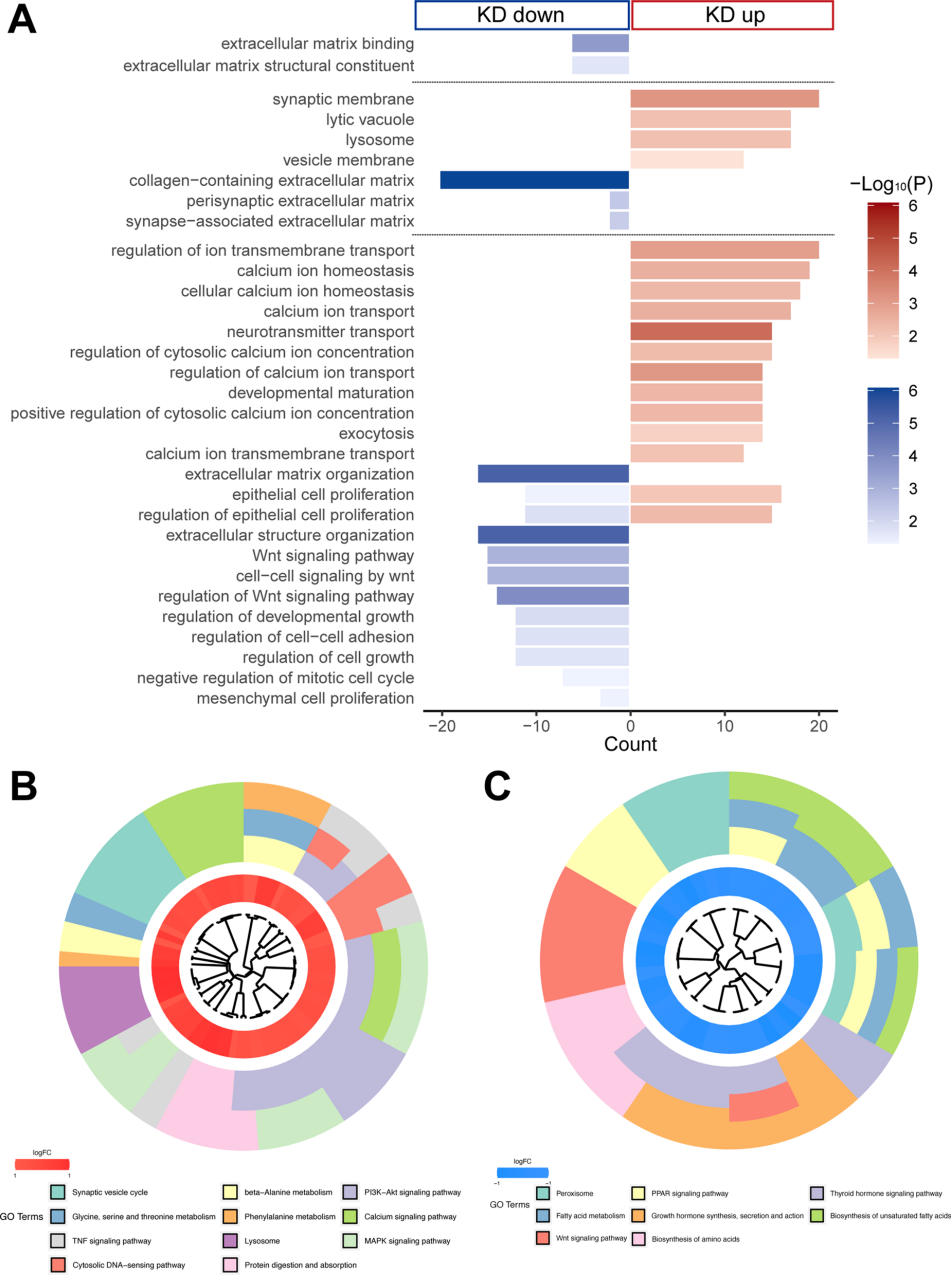
High-throughput sequencing analysis of IFITM3-knockdown cells. **A** GO analysis revealed the expression of genes in IFITM3-knockdown cells. **B**, **C** KEGG Chord analysis showed the signaling pathways enriched in differentially expressed genes (DEGs)

To further explore the regulatory effect of IFITM3 knockdown on the cells, RNA-seq analysis was performed. Among the GO terms, cell membrane and extracellular matrix (ECM)-related, transmembrane transport-related and synaptic transmission enrichment-related terms were the most enriched after knockdown of the IFITM3 gene. These results revealed that genes related to ion transmembrane transport, vacuoles, lysosomes and cellular calcium ion homeostasis were upregulated in the knockdown group, while genes involved in regulation of the ECM, WNT pathway and cell proliferation were significantly downregulated (Fig. 2A), suggesting that the stability of the cell membrane system was seriously damaged and that its material transport function was blocked, ultimately leading to cell death because of constant nutrient deficiency. These findings further reveal how IFITM3 is involved in regulating the proliferation of mNRPCs. The KEGG pathway enrichment assay further revealed that the signaling pathways enriched in the DEGs mainly included the synaptic vesicle cycle, lysosome pathway, calcium signaling pathway, MAPK signaling pathway and other upregulated pathways (Fig. 2B; Additional file 2: Figure S2A), as well as downregulated pathways, such as the WNT, amino acid biosynthesis and fatty acid metabolism pathways (Fig. 2C; Additional file 2: Figure S2B).

Furthermore, GO analysis of the ECM pathway showed that the expression of ion channel-related genes, including the genes encoding calcium (such as CACNB2), potassium (such as KCNB1, KCNF1 and KCNC3) and chloride (such as CLCN1) ion channels, was clearly changed after IFITM3 knockdown (Additional file 2: Figure S2C); additionally, genes that regulate cell proliferation involved in metabolism (such as TNC and MVD) and proliferation-related pathway genes (such as CCND3, STAT1 and IL6) (Additional file 2: Figure S2D) also showed significant changes in expression, indicating that inhibition of IFITM3 expression led to damage to the cell membrane, abnormal material transport, abnormal metabolism of intracellular substances, decreased cell proliferation and ultimately cell death. Taken together, these results indicate that the integrity of the membrane system (including the cell membrane, vacuole, lysosome and ECM) was impaired after IFITM3 knockdown and that cells could no longer survive without sufficient nutrition, eventually leading to cell death.

### Lysosome activation in IFITM3-knockdown cells

**Fig. 3:**
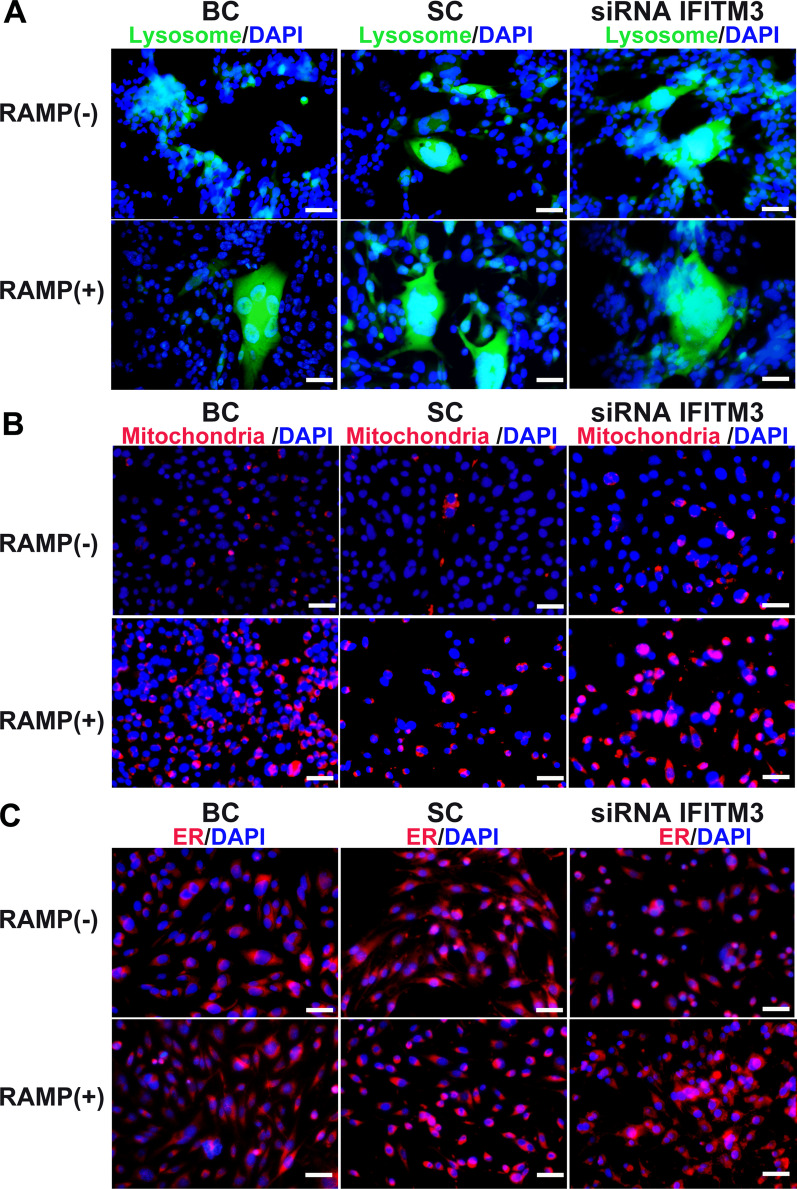
Organelles involved in IFITM3-knockdown cells with or without rapamycin treatment. **A** Fluorescent images showing the localization of lysosomes in cells treated with 50 nM LysoTracker for 30 min. **B** Fluorescence images showing the localization of mitochondria in cells treated with 50 nM MitoTracker for 30 min. **C** Fluorescence images showing localization after the ER in cells treated with 1 μM ER-Tracker for 30 min. Magnification: × 400; scale bar: 50 μm. Abbreviation: BC, blank control; SC, scramble control

Because membrane-related systems were severely damaged after IFITM3 knockdown, we investigated changes in membranous organelles, including the endoplasmic reticulum (ER), mitochondria and lysosomes in the cells. The lysosome is an important site of regulation for mTORC1 signaling, which mainly controls eukaryotic cell growth. We treated cells with 100 nM RAMP, a specific mTOR inhibitor and an autophagy activator, and then treated the cells with 50-nM LysoTracker, 50-nM MitoTracker and 1-μM ER-Tracker for 30 min. Lysosomes showed agglomeration in IFITM3-knockdown cells without RAMP treatment, while lysosomes showed agglomeration in all RAMP treatment groups, but there was no significant difference in IFITM3-knockdown cells with or without RAMP treatment (Fig. 3A). Mitochondria and ERs were increased in IFITM3-knockdown cells treated with RAMP, but there was no significant difference among the groups (Fig. 3B, C), suggesting that mitochondria and ERs were not involved in regulating mNRPC proliferation after IFITM3 knockdown and microautophagy (mA) was not activated.

**Fig. 4:**
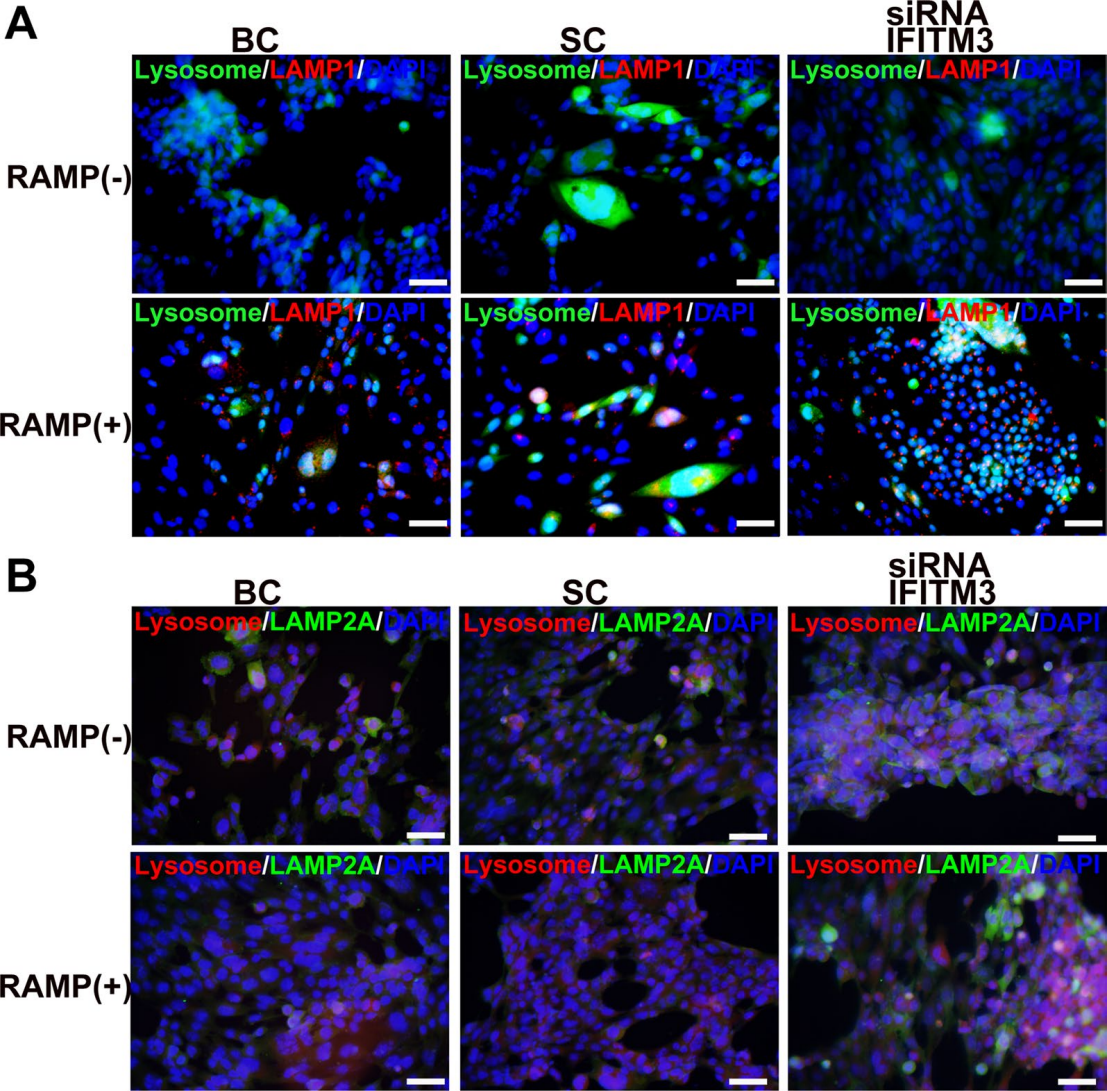
Lysosome activation in IFITM3-knockdown cells treated with or without rapamycin. Immunofluorescence staining of LAMP1 and LAMP2A in cells treated with or without RAMP for 48 h that were labeled with 50 nM LysoTracker for 30 min. **A** The blank control group, **B** Scramble control group and **C** IFITM3-knockdown group. Abbreviation: BC, blank control; SC, scramble control

The lysosome-specific markers lysosome-associated membrane protein 1 (LAMP1) and LAMP2A were detected in RAMP-treated and untreated cells, which showed that the expression of LAMP1 (Fig. 4A) and LAMP2A (Fig. 4B) was significantly increased and accompanied by significantly activated lysosomes in RAMP-treated cells, especially in IFITM3-knockdown cells (Fig. 4A, B), suggesting that the lysosomal system is activated immediately following the destruction of the cell membrane system, eventually causing cell death. These results suggest that IFITM3 in the cell membrane provides the first protective barrier for mNRPCs, and its decreased expression leads to the breakdown of cell homeostasis and initiation of lysosome formation to eliminate damaged cells. Furthermore, there was no significant difference among the IFITM3-knockdown cells with or without RAMP treatment, suggesting that MA was not activated in the cells when the IFITM3 gene was knocked down. However, IFITM3 knockdown led to a cascade of effects causing increased membrane permeability, activation of lysosomes and the CMA pathway, eventually leading to cell death.

### Activation of the CMA pathway when IFITM3 is knocked down

**Fig. 5:**
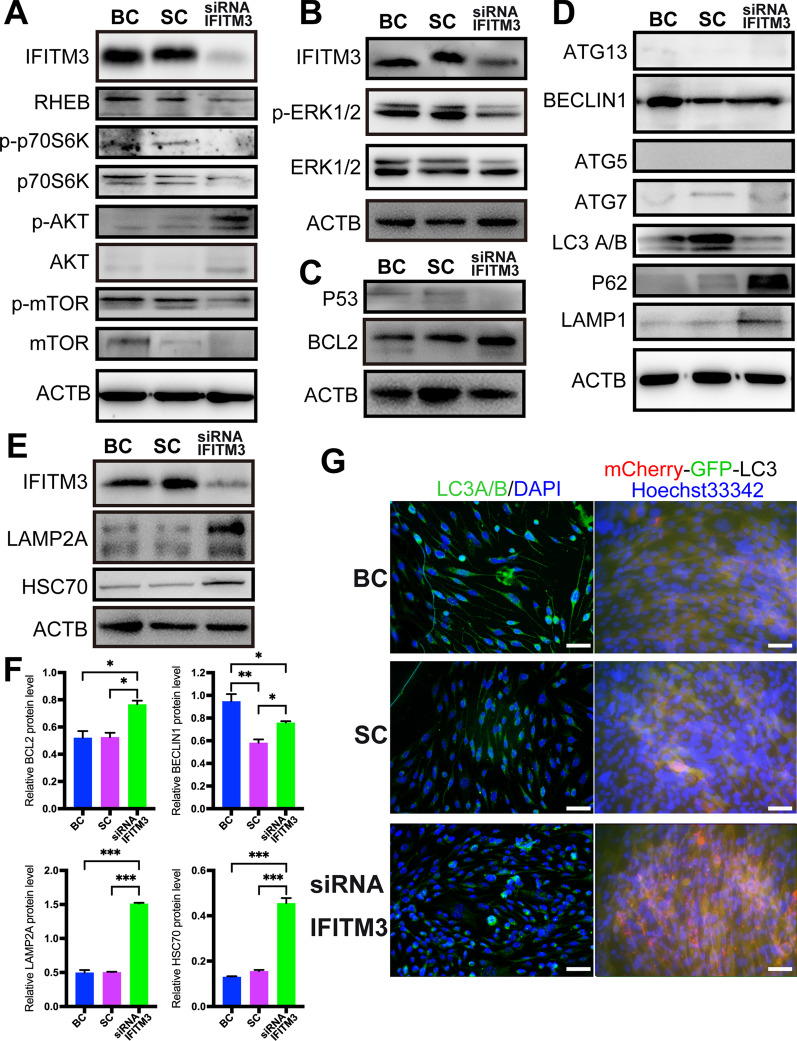
IFITM3 increases the survival and maintains the proliferation of mNRPCs mainly through the CMA signaling pathway. **A** The expression of mTOR pathway-related proteins in cells after IFITM3 knockdown. **B** The expression of ERK1/2 was significantly decreased in IFITM3-knockdown cells. **C** The expression of BCL2 and P53 in IFITM3-knockdown cells. **D** The expression of MA pathway-related proteins in cells after IFITM3 knockdown. **E** CMA was activated, accompanied by increased expression of LAMP2A and HSC70 in cells in which the IFITM3 gene was knocked down. The results are representative of at least three independent experiments, and representative blots are shown. **F** Bar graph showing the relative protein expression levels from the western blot. **G** Results of IHC analysis of LC3A/B and fluorescent images showing mCherry-GFP-LC3 expression after IFITM3 knockdown used to assay autophagic flux in the cells. Magnification: × 400; scale bar: 50 μm

Cell viability was significantly inhibited and eventually caused cell death after IFITM3 knockdown. Therefore, signal pathways regulating cell proliferation including mTOR pathway and MAPK pathway were detected, the data showed no significant activation of the mTOR pathway (Fig. 5A), but obviously decreased expression of ERK1/2 among the groups (Fig. 5B), suggesting that the downregulation of IFITM3 resulted in decreased cell survival and that the ability of the cells to proliferate was severely reduced.

Then, the results of western blot analysis showed significantly increased expression of the antiapoptotic protein BCL2 (Fig. 5C, F) and decreased expression of P53 (Fig. 5C) in IFITM3-knockdown cells, which suggested that the apoptotic pathway was not activated when IFITM3 was knocked down. Additionally, we examined apoptotic cells by staining the cells with Annexin V-PE, the results showed no obvious apoptosis in IFITM3-knockdown cells (Additional file 3: Fig. S3A–C), and no apoptotic bodies were detected by Hoechst 33342 staining (Additional file 3: Fig. S3D–F), which consistent with WB results.

Next, the further investigation focused on the MA and CMA pathways. The expression of MA pathway-related proteins is complicated, there was no significant change in ATG13, BECLIN1, ATG5, ATG7 and LC3A/B, but P62 and LAMP1 were significantly upregulated (Fig. 5D, F), the increased LAMP1 suggested the formation of autophagy lysosomes, and the results suggested that the MA pathway was not activated after IFITM3 knockdown. Therefore, MA is not the pathway of this regulatory effect.

However, the expression of LAMP2A and HSC70 (Fig. 5E, F) was significantly upregulated, which indicated and confirmed the activation of CMA in the cells after IFITM3 knockdown. Although the mRNA expression (Additional file 4: Fig. S4) was not consistent with the protein expression after knockdown IFITM3 for 48 h, the specific reasons need to be further studied in the future. To further confirm the activation of MA or CMA, the expression of LC3A/B in IFITM3-knockdown cells was analyzed with immunohistochemical (IHC) first, the results showed that there was no significant difference in the cells (Fig. 5G). Then, the generation of autophagic flux was examined by constructing stable LC3-GFP-mCherry-expressing mNRPCs. The results showed that no autophagic flux was generated in the cells (Fig. 5G; Additional file 5: Fig. S5).

**Fig. 6:**
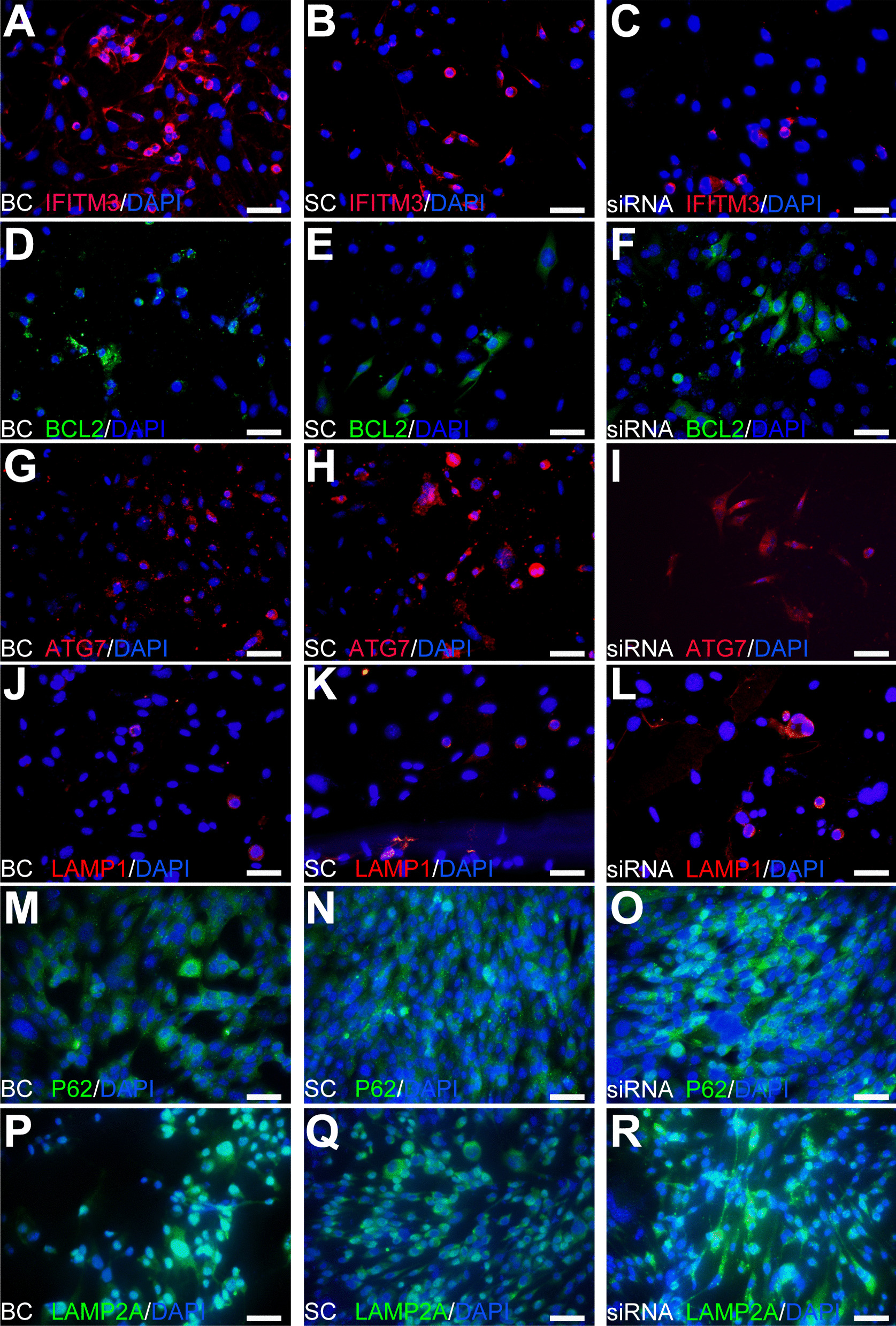
IHC assay upon IFITM3 knockdown. **A**–**C** Immunofluorescence staining of IFITM3 in the different groups. **D**–**F** Expression of the classic apoptosis pathway-related protein BCL2. **G**–**O** MA pathway-related proteins: ATG7, LAMP1 and P62. **P–R** The expression of the CMA pathway protein LAMP2A was consistent with the WB results. The results are representative of at least three independent experiments, and representative views are shown. Magnification: × 400; scale bar: 50 μm. Abbreviation: BC, blank control; SC, scramble control

Some of the above results were consistent with IHC results. When IFITM3 was knocked down (Fig. 6A–C), the results of IHC analysis showed slightly increased expression of BCL2 (Fig. 6D–F) and no significant changes in ATG7 expression (Fig. 6G–I), while the expression of LAMP1 (Fig. 6J–L), P62 (Fig. 6M–O) and LAMP2A (Fig. 6P–R) was significantly changed. The data were consistent with the WB results and IHC data during lysosome activation, indicating no activation of MA or the apoptotic pathway, while the CMA pathway was activated in IFITM3-knockdown cells. According to the above data, we believe that IFITM3 regulates cell viability mainly by regulating the CMA pathway, suggesting that the IFITM3 gene has a protective function that increases progenitor cell survival and self-renewal.

## Discussion

Stem cell-related cell therapies have developed rapidly around the world. In the field of cellular ophthalmology therapy, safety data have emerged from several phase I/II clinical trials of cell transplantation with retinal pigment epithelial (RPE) cells or MSCs [25–35]; however, some issues, such as host-graft rejection, validation over too short a duration (less than 12 months) and a lack of clinical significance, remain. Although RPC transplantation is considered a very promising approach [2, 36–38], its application is limited because of insufficient donor cells. Therefore, the need to obtain enough seed cells has become urgent. Previously, we established mNRPCs that can be cultured long-term in vitro and maintain their self-renewal properties based on the combination of bFGF and CHIR99021 [3]. In further biological studies, RNA-seq data showed the high expression of some genes that are expressed at low levels in adult retinal tissue, such as IFITM3, an intrinsic host factor with extensive function in the organism, including antiviral function [39, 40], immune function [41–44] and germ cell specification [45] that is localized in several cellular components, including the apical part of the cell, cell surface and endolysosomal membrane. Currently, limited reports on the role of IFITM3 in stem cells are available, and its specific function in the field is still unclear. Here, we studied the function of the IFITM3 gene in mNRPCs by knocking it down, and we focused on investigating the changes in the autophagy pathway, which is very important to cell death and homeostasis. In mNRPCs, we found that chaperone-mediated autophagy (CMA) plays a very important function in regulating retinal progenitor cell homeostasis.

Our analysis provides a series of studies of signaling pathways involved in regulating cell growth and death, and mNRPCs showed significantly decreased cell viability and proliferation after IFITM3 knockdown. The significantly decreased expression of CCND1, a key molecule in the regulation of cell cycle progression from G1 to S phase, suggests that IFITM3 affects the proliferation of mNRPCs by regulating cell division. In addition, the expression of ERK1/2 was decreased accordingly after IFITM3 knockdown, indicating that IFITM3 regulates cell proliferation and may be associated with the MAPK pathway. And the apoptotic pathway was not activated when IFITM3 was knocked down. Further detection revealed that the CMA pathway was activated with high expression of HSC70 and LAMP2A, although the mRNA expression was not consistent with the protein expression. It has been reported that CMA began to increase at 8 h after serum starvation and peaked at 36 h, lasting for 3 days [46]. Overall, our work provides a basis for understanding how IFITM3 regulates RPC proliferation and homeostasis.

As one of the important members of the autophagy family, macroautophagy (MA), mitophagy (mA) [47–49] and CMA [49–52], CMA starts with HSC70, and HSC70 targets cargo proteins and then binds to LAMP2A to translocate the proteins into the lysosomal lumen. CMA activity is detectable under basal conditions in most cells, but overactivation occurs during stress, such as nutrient deprivation [53] and lipid overload [54, 55]. Consistently, our IFITM3 knockdown cell model demonstrated that IFITM3 maintains RPC cell homeostasis by stabilizing the cell membrane or cellular membranous organelles. Consistent with the LAMP2A immunolabeling data, WB and lysosome activation assays confirmed the overactivation of CMA. Moreover, RNA-seq data showed significant changes in the membrane system, and GO and KEGG analysis of the RNA-seq results suggested that IFITM3 is involved in regulating mNRPC proliferation by changing the structure and function of the cell membrane. Additionally, lysosomes also showed a marked increase in abundance in this abnormal membrane system. Our results suggest that CMA activation is due to the instability of the cell membrane system, lysosomal activation and accumulation caused by IFIMT3 knockdown.

Knowing that lysosomes are a key factor in autophagy and important for regulating mTORC1 signaling [56–59], the main regulator of autophagy, especially MA, we studied changes in lysosomes and the autophagy pathway after RAMP treatment when IFITM3 was knocked down. An increase in the expression of LAMP1 and LAMP2A in knockdown cells suggests the activation of lysosomes, consistent with the LysoTracker results. We believe that IFITM3 regulates cell proliferation mainly by activating the CMA pathway, and lysosomes are the key member of this regulatory process. Some similarity exists between our data and another study that observed protein quality control in quiescent steady-state hematopoietic stem cells (HSCs) of CMA [52].

Taken together, this evidence suggests a protective role for IFITM3 interactions in mediating self-renewal and cellular homeostasis in mNRPCs. The decreased expression of IFITM3 in mNRPCs resulted in the destruction of the cell membrane system and activation of lysosomes, a key factor in the autophagy pathway. Similar to our findings, IFITM3 has been reported to shuttle virus particles directly to lysosomes [60]. In mNRPCs, lysosomes were activated to remove the stress damage in cells caused by IFITM3 knockout and accompanied by activation of the CMA pathway, and continuous overactivation of CMA caused cell death.

Overall, the study provides novel insights into the role of IFITM3 in RPCs, such as solving the problem of maintaining self-renewal in vitro by regulating the CMA pathway or attempting to activate quiescent in vivo RPC cells to repair damaged retinas in retinal degenerative diseases.

## Conclusion

In this report, we found that IFITM3 plays an important role in RPC proliferation by activating the CMA pathway, suggesting that the low-level activation of CMA is important in maintaining the homeostasis of RPCs, while the overactivation of CMA leads to cell death.

## Supplementary Information

**Additional file 1.** **Figure S1:** Cell morphology and expression of IFITM3 of mNRPCs after knockdown with siRNA for 48 h.

**Additional file 2.** **Figure S2:** High-throughput sequencing analysis of IFITM3-knockdown cells.

**Additional file 3.** **Figure S3:** Cell apoptosis assay after IFITM3-knockdown for 48 h.

**Additional file 4.** **Figure S4:** qRT–PCR assay of LAMP2A and HSC70 expression in mNRPCs after knockdown IFITM3 for 48 h.

**Additional file 5.** **Figure S5:** Fluorescent images showing mCherry-GFP-LC3 expression after IFITM3 knockdown used to assay autophagic flux in the cells.

**Additional file 6.** Supplementary Material.

## Data Availability

RNA-seq data generated in the study can be accessed at the Gene Expression Omnibus under accession code GSE176472(https://www.ncbi.nlm.nih.gov/geo/query/acc.cgi?acc=GSE176472). The original gel images of the western blot were supplied in the supplementary material (supplementary material Original gel images). All other data are included within the article and its additional files.

## Abbreviations

BC: Blank control
CCND1: Cyclin D1
CMA: Chaperone-mediated autophagy
DEG: Differentially expressed gene
ECM: Extracellular matrix
ER: Endoplasmic reticulum
GO: Gene ontology
IFITM3: Interferon-induced transmembrane protein 3
KEGG: Kyoto encyclopedia of genes and genomes
LAMP1: Lysosome-associated membrane protein 1
mA: Microautophagy
mNRPC: Mouse neural retinal progenitor cell
MSC: Mesenchymal stem cells
OD: Optical density
PFA: Paraformaldehyde
RAMP: Rapamycin
RPC: Retinal progenitor cell
SC: Scramble control
UMAP: Uniform manifold approximation and projection

## References

[1] Wang Z, Gao F, Zhang M, Zheng Y, Zhang F, Xu L, Cao L, He W (2020). Intravitreal injection of human retinal progenitor cells for treatment of retinal degeneration. Med Sci Monit.

[2] Liu Y, Chen SJ, Li SY, Qu LH, Meng XH, Wang Y, Xu HW, Liang ZQ, Yin ZQ (2017). Long-term safety of human retinal progenitor cell transplantation in retinitis pigmentosa patients. Stem Cell Res Ther.

[3] Jin C, Ou Q, Li Z, Wang J, Zhang J, Tian H, Xu JY, Gao F, Lu L, Xu GT (2018). The combination of bFGF and CHIR99021 maintains stable self-renewal of mouse adult retinal progenitor cells. Stem Cell Res Ther.

[4] Butler A, Hoffman P, Smibert P, Papalexi E, Satija R (2018). Integrating single-cell transcriptomic data across different conditions, technologies, and species. Nat Biotechnol.

[5] Brass AL, Huang IC, Benita Y, John SP, Krishnan MN, Feeley EM, Ryan BJ, Weyer JL, van der Weyden L, Fikrig E (2009). The IFITM proteins mediate cellular resistance to influenza A H1N1 virus, West Nile virus, and dengue virus. Cell.

[6] Schoggins JW, Wilson SJ, Panis M, Murphy MY, Jones CT, Bieniasz P, Rice CM (2011). A diverse range of gene products are effectors of the type I interferon antiviral response. Nature.

[7] Weidner JM, Jiang D, Pan XB, Chang J, Block TM, Guo JT (2010). Interferon-induced cell membrane proteins, IFITM3 and tetherin, inhibit vesicular stomatitis virus infection via distinct mechanisms. J Virol.

[8] Yount JS, Moltedo B, Yang YY, Charron G, Moran TM, Lopez CB, Hang HC (2010). Palmitoylome profiling reveals S-palmitoylation-dependent antiviral activity of IFITM3. Nat Chem Biol.

[9] Galvin HD, Husain M (2019). Influenza A virus-induced host caspase and viral PA-X antagonize the antiviral host factor, histone deacetylase 4. J Biol Chem.

[10] Sun Q, Lei N, Lu J, Gao RB, Li Z, Liu LQ, Sun Y, Guo JF, Wang DY, Shu YL (2020). Interferon-induced transmembrane protein 3 prevents acute influenza pathogenesis in mice. Biomed Environ Sci.

[11] Saitou M, Payer B, Lange UC, Erhardt S, Barton SC, Surani MA (2003). Specification of germ cell fate in mice. Philos Trans R Soc Lond B Biol Sci.

[12] Tanaka SS, Nagamatsu G, Tokitake Y, Kasa M, Tam PP, Matsui Y (2004). Regulation of expression of mouse interferon-induced transmembrane protein like gene-3, Ifitm3 (mil-1, fragilis), in germ cells. Dev Dyn.

[13] Tanaka SS, Yamaguchi YL, Tsoi B, Lickert H, Tam PP (2005). IFITM/Mil/fragilis family proteins IFITM1 and IFITM3 play distinct roles in mouse primordial germ cell homing and repulsion. Dev Cell.

[14] Jiang LQ, Xia T, Hu YH, Sun MS, Yan S, Lei CQ, Shu HB, Guo JH, Liu Y (2018). IFITM3 inhibits virus-triggered induction of type I interferon by mediating autophagosome-dependent degradation of IRF3. Cell Mol Immunol.

[15] Yount JS, Karssemeijer RA, Hang HC (2012). S-palmitoylation and ubiquitination differentially regulate interferon-induced transmembrane protein 3 (IFITM3)-mediated resistance to influenza virus. J Biol Chem.

[16] Ohsumi Y (1999). Molecular mechanism of autophagy in yeast, *Saccharomyces cerevisiae*. Philos Trans R Soc Lond B Biol Sci.

[17] Goyal A, Neill T, Owens RT, Schaefer L, Iozzo RV (2014). Decorin activates AMPK, an energy sensor kinase, to induce autophagy in endothelial cells. Matrix Biol.

[18] Ashoor R, Yafawi R, Jessen B, Lu S (2013). The contribution of lysosomotropism to autophagy perturbation. PLoS ONE.

[19] Brenner C, Galluzzi L, Kepp O, Kroemer G (2013). Decoding cell death signals in liver inflammation. J Hepatol.

[20] Kim MJ, Febbraro D, Farkona S, Gillmore T, Son JE, Regeenes R, Chang HH, Pollock-Tahiri E, Yang J, Park YJ (2021). Distinct roles of UVRAG and EGFR signaling in skeletal muscle homeostasis. Mol Metab.

[21] Tseng WC, Lee PY, Tsai MT, Chang FP, Chen NJ, Chien CT, Hung SC, Tarng DC (2021). Hypoxic mesenchymal stem cells ameliorate acute kidney ischemia-reperfusion injury via enhancing renal tubular autophagy. Stem Cell Res Ther.

[22] Tang J, Ye Z, Liu Y, Zhou M, Huang L, Mo Q, Su X, Qin C (2021). Autophagy-deficiency in bone marrow mononuclear cells from patients with myasthenia gravis: a possible mechanism of pathogenesis. Int J Neurosci.

[23] Savio-Silva C, Soinski-Sousa PE, Simplicio-Filho A, Bastos RMC, Beyerstedt S, Rangel EB (2021). Therapeutic potential of mesenchymal stem cells in a pre-clinical model of diabetic kidney disease and obesity. Int J Mol Sci.

[24] Clark BS, Stein-O'Brien GL, Shiau F, Cannon GH, Davis-Marcisak E, Sherman T, Santiago CP, Hoang TV, Rajaii F, James-Esposito RE (2019). Single-cell RNA-Seq analysis of retinal development identifies NFI factors as regulating mitotic exit and late-born cell specification. Neuron.

[25] Algvere PV, Berglin L, Gouras P, Sheng Y, Kopp ED (1997). Transplantation of RPE in age-related macular degeneration: observations in disciform lesions and dry RPE atrophy. Graefes Arch Clin Exp Ophthalmol.

[26] da Cruz L, Fynes K, Georgiadis O, Kerby J, Luo YH, Ahmado A, Vernon A, Daniels JT, Nommiste B, Hasan SM (2018). Phase 1 clinical study of an embryonic stem cell-derived retinal pigment epithelium patch in age-related macular degeneration. Nat Biotechnol.

[27] Humayun MS (2000). Human neural retinal transplantation. Invest Ophthalmol Vis Sci.

[28] Radtke ND, Aramant RB, Petry HM, Green PT, Pidwell DJ, Seiler MJ (2008). Vision improvement in retinal degeneration patients by implantation of retina together with retinal pigment epithelium. Am J Ophthalmol.

[29] Schwartz SD, Hubschman JP, Heilwell G, Franco-Cardenas V, Pan CK, Ostrick RM, Mickunas E, Gay R, Klimanskaya I, Lanza R (2012). Embryonic stem cell trials for macular degeneration: a preliminary report. Lancet.

[30] Schwartz SD, Regillo CD, Lam BL, Eliott D, Rosenfeld PJ, Gregori NZ, Hubschman JP, Davis JL, Heilwell G, Spirn M (2015). Human embryonic stem cell-derived retinal pigment epithelium in patients with age-related macular degeneration and Stargardt's macular dystrophy: follow-up of two open-label phase 1/2 studies. Lancet.

[31] Song WK, Park KM, Kim HJ, Lee JH, Choi J, Chong SY, Shim SH, Del Priore LV, Lanza R (2015). Treatment of macular degeneration using embryonic stem cell-derived retinal pigment epithelium: preliminary results in Asian patients. Stem Cell Rep.

[32] Siqueira RC, Messias A, Voltarelli JC, Scott IU, Jorge R (2011). Intravitreal injection of autologous bone marrow-derived mononuclear cells for hereditary retinal dystrophy: a phase I trial. Retina.

[33] Siqueira RC, Messias A, Voltarelli JC, Messias K, Arcieri RS, Jorge R (2013). Resolution of macular oedema associated with retinitis pigmentosa after intravitreal use of autologous BM-derived hematopoietic stem cell transplantation. Bone Marrow Transplant.

[34] Siqueira RC, Messias A, Messias K, Arcieri RS, Ruiz MA, Souza NF, Martins LC, Jorge R (2015). Quality of life in patients with retinitis pigmentosa submitted to intravitreal use of bone marrow-derived stem cells (Reticell -clinical trial). Stem Cell Res Ther.

[35] Satarian L, Nourinia R, Safi S, Kanavi MR, Jarughi N, Daftarian N, Arab L, Aghdami N, Ahmadieh H, Baharvand H (2017). Intravitreal injection of bone marrow mesenchymal stem cells in patients with advanced retinitis pigmentosa; a safety study. J Ophthalmic Vis Res.

[36] Hendrickson A, Bumsted-O'Brien K, Natoli R, Ramamurthy V, Possin D, Provis J (2008). Rod photoreceptor differentiation in fetal and infant human retina. Exp Eye Res.

[37] Gu P, Harwood LJ, Zhang X, Wylie M, Curry WJ, Cogliati T (2007). Isolation of retinal progenitor and stem cells from the porcine eye. Mol Vis.

[38] Tang Z, Jiang F, Zhang Y, Zhang Y (2019). Mussel-inspired injectable hydrogel and its counterpart for actuating proliferation and neuronal differentiation of retinal progenitor cells. Biomaterials.

[39] Li C, Du S, Tian M, Wang Y, Bai J, Tan P, Liu W, Yin R, Wang M, Jiang Y (2018). The host restriction factor interferon-inducible transmembrane protein 3 inhibits vaccinia virus infection. Front Immunol.

[40] Smith SE, Busse DC, Binter S, Weston S, Diaz Soria C, Laksono BM, Clare S, Van Nieuwkoop S, Van den Hoogen BG, Clement M (2019). Interferon-induced transmembrane protein 1 restricts replication of viruses that enter cells via the plasma membrane. J Virol.

[41] Wee YS, Roundy KM, Weis JJ, Weis JH (2012). Interferon-inducible transmembrane proteins of the innate immune response act as membrane organizers by influencing clathrin and v-ATPase localization and function. Innate Immun.

[42] Verrier ER, Colpitts CC, Bach C, Heydmann L, Zona L, Xiao F, Thumann C, Crouchet E, Gaudin R, Sureau C (2016). Solute carrier NTCP regulates innate antiviral immune responses targeting hepatitis C virus infection of hepatocytes. Cell Rep.

[43] Campbell RA, Schwertz H, Hottz ED, Rowley JW, Manne BK, Washington AV, Hunter-Mellado R, Tolley ND, Christensen M, Eustes AS (2019). Human megakaryocytes possess intrinsic antiviral immunity through regulated induction of IFITM3. Blood.

[44] Nutsford AN, Galvin HD, Ahmed F, Husain M (2019). The Class IV human deacetylase, HDAC11, exhibits anti-influenza A virus properties via its involvement in host innate antiviral response. Cell Microbiol.

[45] Young JC, Dias VL, Loveland KL (2010). Defining the window of germline genesis in vitro from murine embryonic stem cells. Biol Reprod.

[46] Koga H, Martinez-Vicente M, Macian F, Verkhusha VV, Cuervo AM (2011). A photoconvertible fluorescent reporter to track chaperone-mediated autophagy. Nat Commun.

[47] Li M, Guo J, Wang H, Li Y (2021). Involvement of mitochondrial dynamics and mitophagy in sevoflurane-induced cell toxicity. Oxid Med Cell Longev.

[48] Lizama BN, Chu CT (2021). Neuronal autophagy and mitophagy in Parkinson's disease. Mol Asp Med.

[49] Zheng J, Wei S, Xiao T, Li G (2021). LC3B/p62-mediated mitophagy protects A549 cells from resveratrol-induced apoptosis. Life Sci.

[50] Tokarchuk I, Janser FA, Schlafli AM, Pinto MT, Humbert M, Niklaus NJ, Berezowska S, Langer R, Tschan MP (2021). Increased LAMP2A levels correlate with a shorter disease-free survival of HER2 negative breast cancer patients and increased breast cancer cell viability. Biochem Biophys Res Commun.

[51] Xu X, Sun Y, Cen X, Shan B, Zhao Q, Xie T, Wang Z, Hou T, Xue Y, Zhang M (2021). Metformin activates chaperone-mediated autophagy and improves disease pathologies in an Alzheimer disease mouse model. Protein Cell.

[52] Dong S, Wang Q, Kao YR, Diaz A, Tasset I, Kaushik S, Thiruthuvanathan V, Zintiridou A, Nieves E, Dzieciatkowska M (2021). Chaperone-mediated autophagy sustains haematopoietic stem-cell function. Nature.

[53] Cuervo AM, Knecht E, Terlecky SR, Dice JF (1995). Activation of a selective pathway of lysosomal proteolysis in rat liver by prolonged starvation. Am J Physiol.

[54] Kaushik S, Cuervo AM (2015). Degradation of lipid droplet-associated proteins by chaperone-mediated autophagy facilitates lipolysis. Nat Cell Biol.

[55] Rodriguez-Navarro JA, Kaushik S, Koga H, Dall'Armi C, Shui G, Wenk MR, Di Paolo G, Cuervo AM (2012). Inhibitory effect of dietary lipids on chaperone-mediated autophagy. Proc Natl Acad Sci USA.

[56] Atwood DJ, Pokhrel D, Brown CN, Holditch SJ, Bachu DM, Thorburn A, Hopp K, Edelstein CL (2020). Increased mTOR and suppressed autophagic flux in the heart of a hypomorphic Pkd1 mouse model of autosomal dominant polycystic kidney disease. Cell Signal.

[57] Sironi J, Aranda E, Nordstrom LU, Schwartz EL (2019). Lysosome membrane permeabilization and disruption of the molecular target of rapamycin (mTOR)-lysosome interaction are associated with the inhibition of lung cancer cell proliferation by a chloroquinoline analog. Mol Pharmacol.

[58] Chen Y, Xu S, Wang N, Ma Q, Peng P, Yu Y, Zhang L, Ying Z, Wang H (2019). Dynasore suppresses mTORC1 activity and induces autophagy to regulate the clearance of protein aggregates in neurodegenerative diseases. Neurotox Res.

[59] Hong Z, Pedersen NM, Wang L, Torgersen ML, Stenmark H, Raiborg C (2017). PtdIns3P controls mTORC1 signaling through lysosomal positioning. J Cell Biol.

[60] Spence JS, He R, Hoffmann HH, Das T, Thinon E, Rice CM, Peng T, Chandran K, Hang HC (2019). IFITM3 directly engages and shuttles incoming virus particles to lysosomes. Nat Chem Biol.

