# Supplementary figures and images for: Chaperone-mediated autophagy plays an important role in regulating retinal progenitor cell homeostasis
Source: Stem Cell Res Ther. 2022 Apr 1;13:136. doi: 10.1186/s13287-022-02809-z (PMC8973999; doi:10.1186/s13287-022-02809-z)

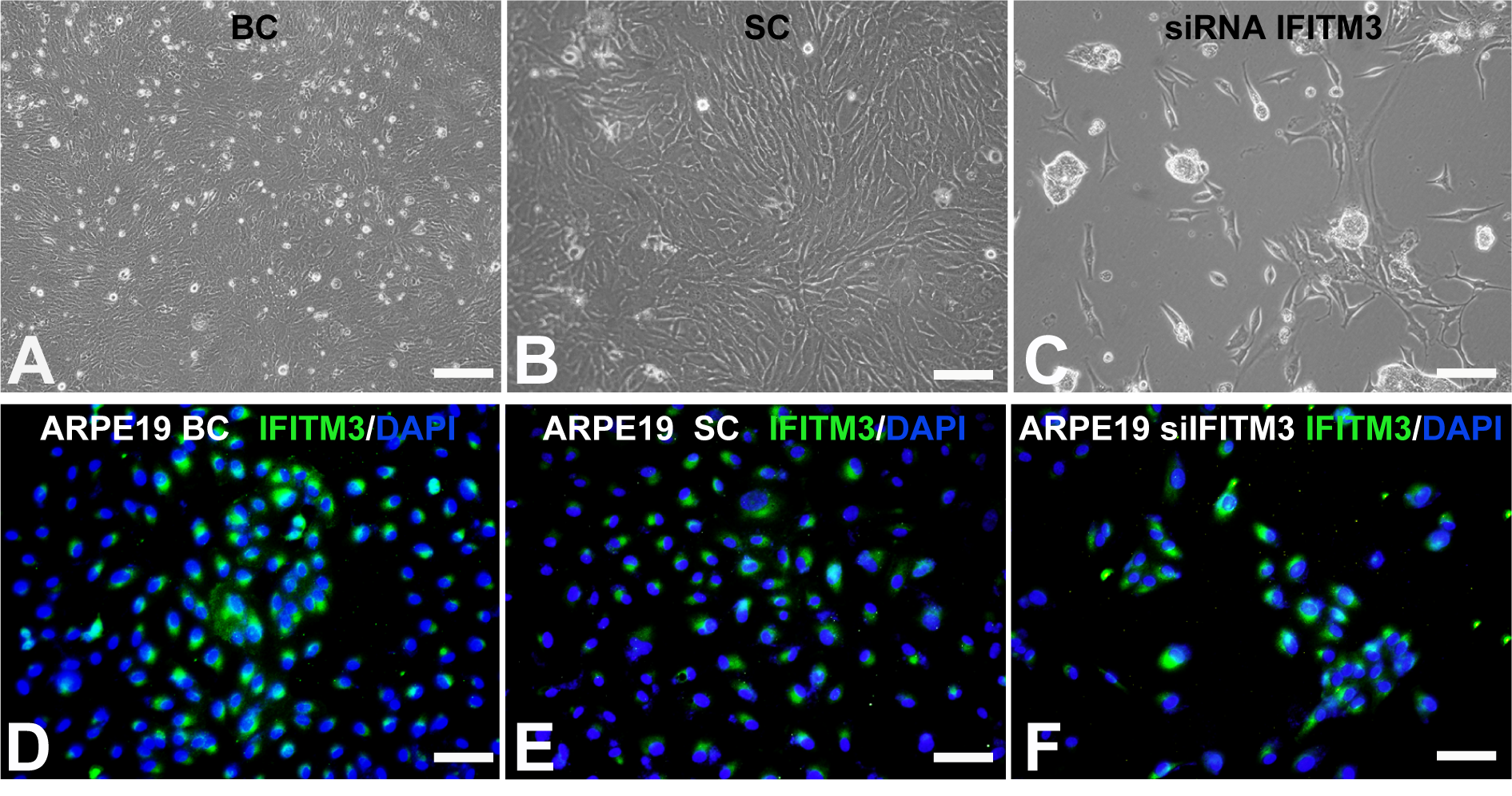

Supplement: Supplementary file 1 — Additional file 1. Figure S1: Cell morphology and expression of IFITM3 of mNRPCs after knockdown with siRNA for 48 h. [file 13287_2022_2809_MOESM1_ESM.tif]

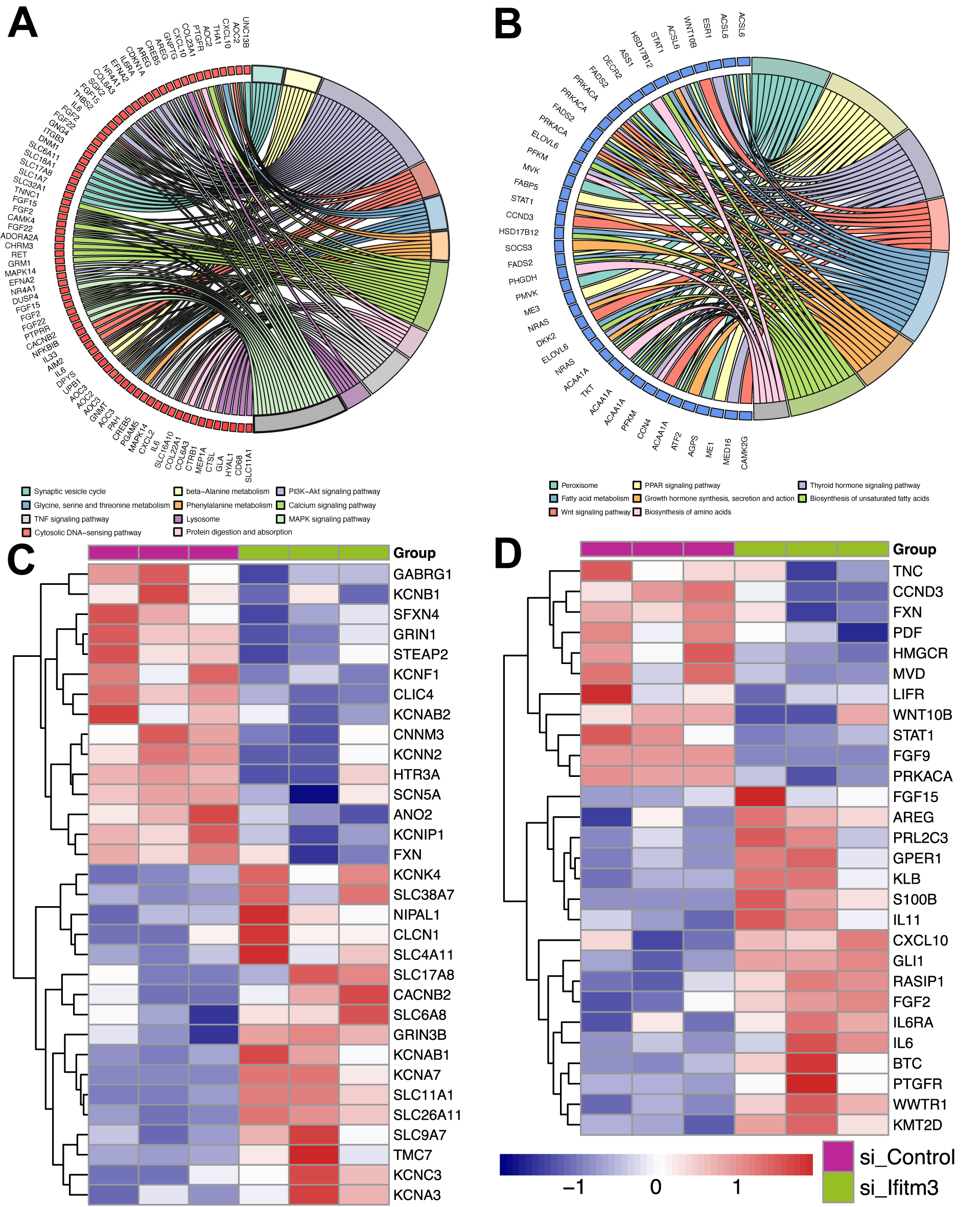

Supplement: Supplementary file 2 — Additional file 2. Figure S2: High-throughput sequencing analysis of IFITM3-knockdown cells. [file 13287_2022_2809_MOESM2_ESM.tif]

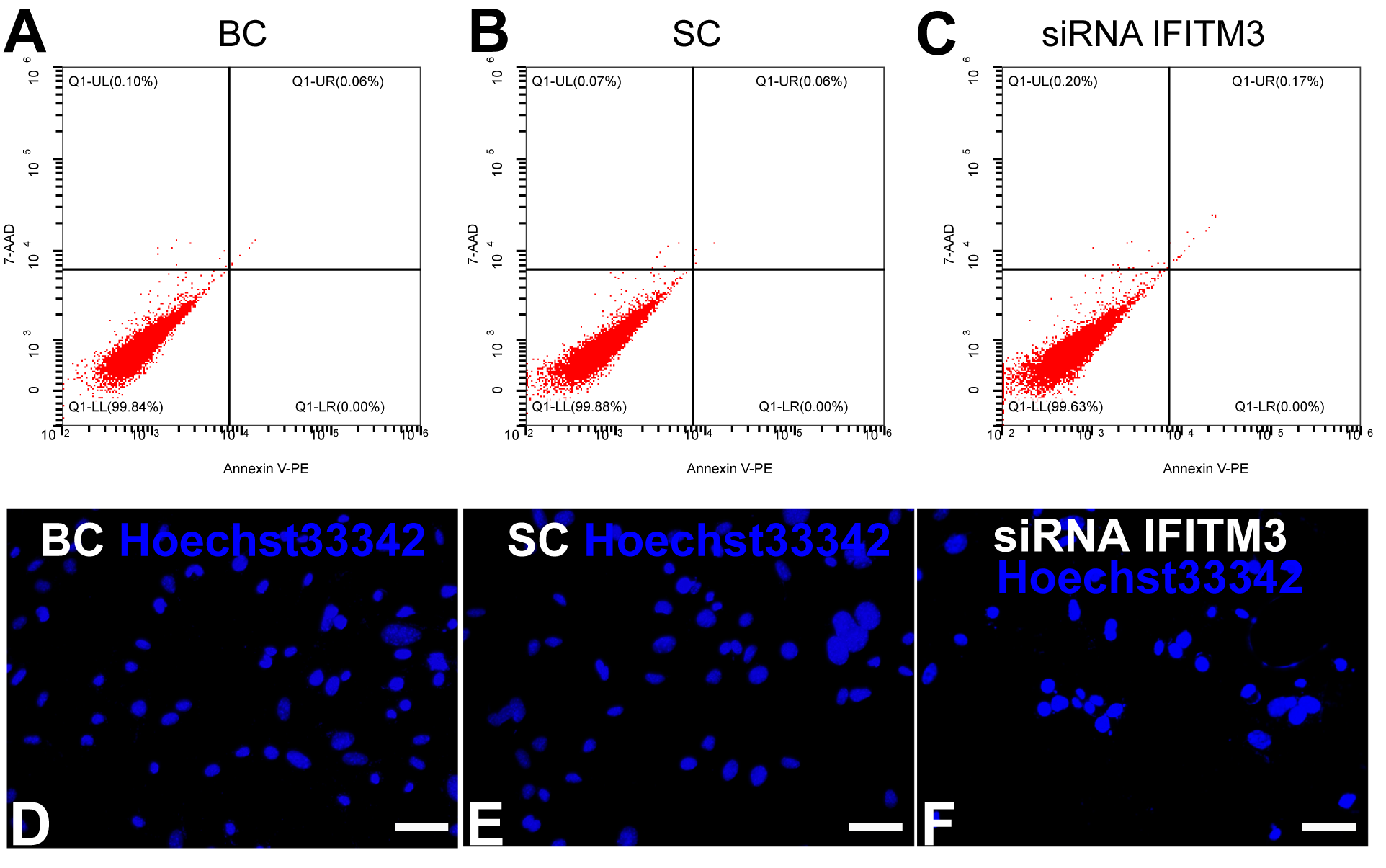

Supplement: Supplementary file 3 — Additional file 3. Figure S3: Cell apoptosis assay after IFITM3-knockdown for 48 h. [file 13287_2022_2809_MOESM3_ESM.tif]

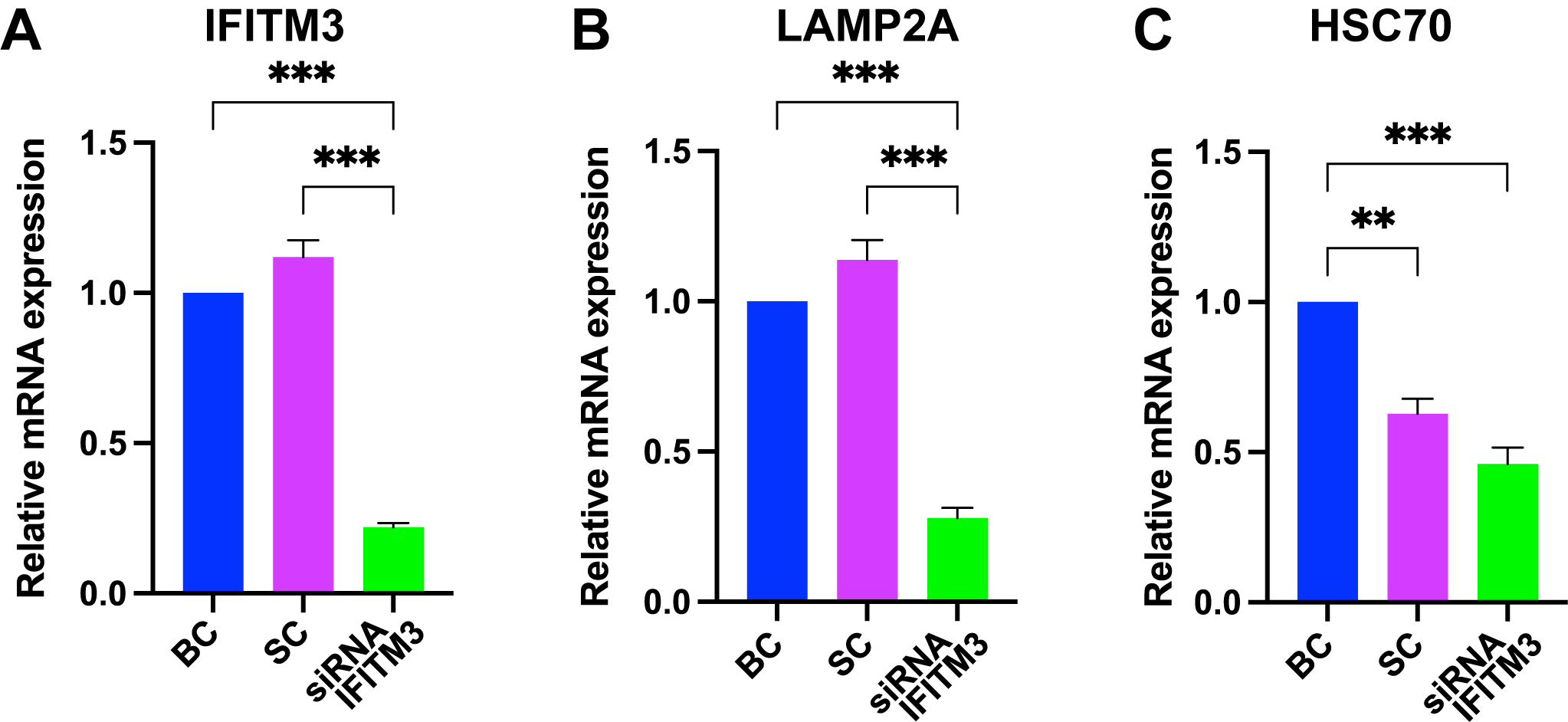

Supplement: Supplementary file 4 — Additional file 4. Figure S4: qRT–PCR assay of LAMP2A and HSC70 expression in mNRPCs after knockdown IFITM3 for 48 h. [file 13287_2022_2809_MOESM4_ESM.tif]

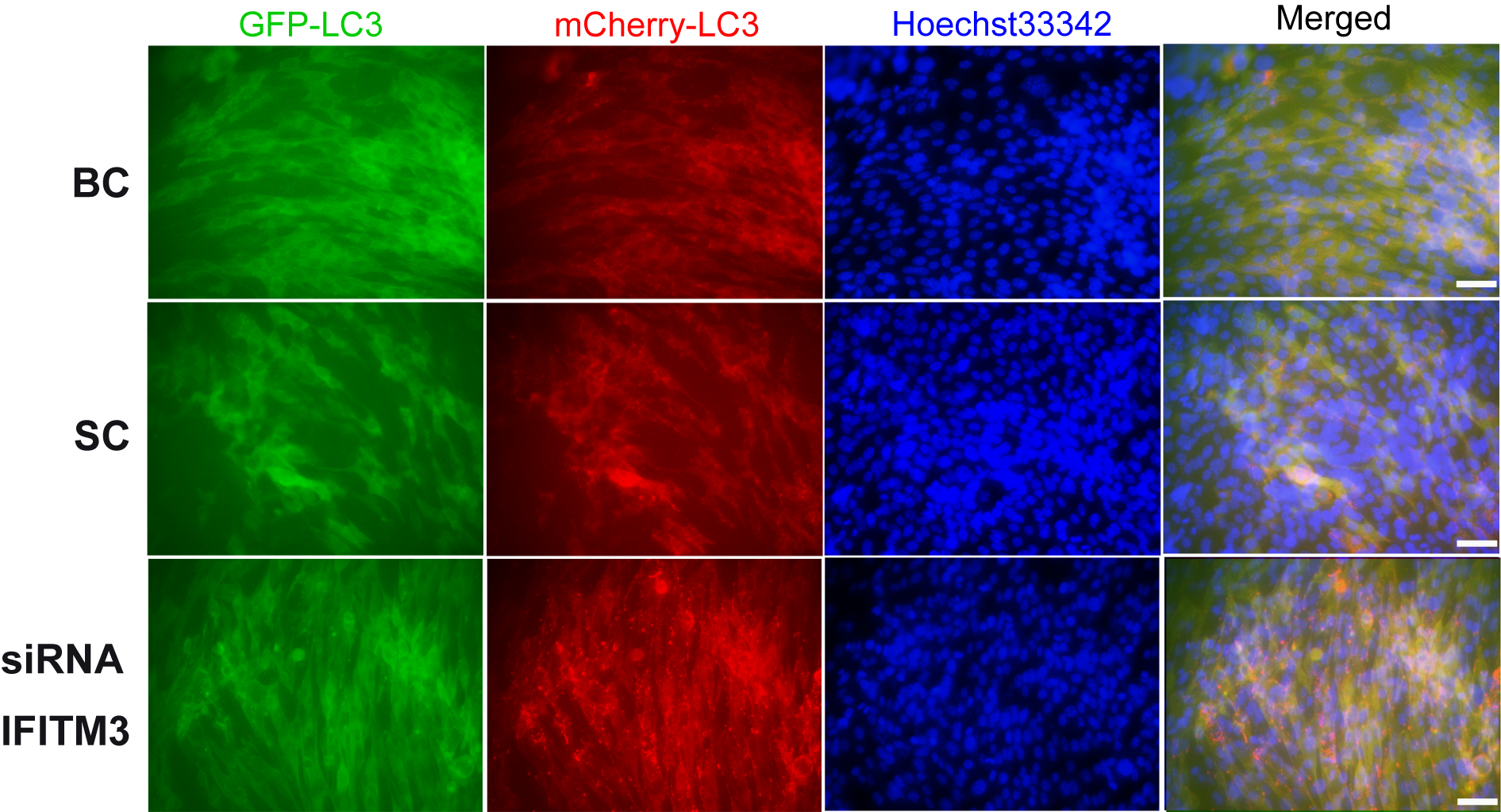

Supplement: Supplementary file 5 — Additional file 5. Figure S5: Fluorescent images showing mCherry-GFP-LC3 expression after IFITM3 knockdown used to assay autophagic flux in the cells. [file 13287_2022_2809_MOESM5_ESM.tif]
