# Supplementary material for: Chaperone-mediated autophagy plays an important role in regulating retinal progenitor cell homeostasis
Source: Stem Cell Res Ther. 2022 Apr 1;13:136. doi: 10.1186/s13287-022-02809-z (PMC8973999; doi:10.1186/s13287-022-02809-z)
Supplement: Supplementary file 6 — Additional file 6. Supplementary Material. [file 13287_2022_2809_MOESM6_ESM.docx]

Supplementary Material

# Supplementary methods

**1.1 Cell apoptosis assay**

For the apoptotic cell assay, after 48 h of transfection with IFITM3 siRNA, Annexin V-PE (Yeasen, China) was added to the cells for 15 min at room temperature in dark. The cells were washed 3 times with PBS (Sangon Biotech, China), then assayed with CytoFLEX LX system (BECKMAN COULTER, USA).

# Supplementary figures and tables

**2.1 Supplementary Table 1: Primers used in the RNA interference experiment and qPCR**

| **Genes** | **Forward (5′-3′)** | **Reverse (5′-3′)** |
| --- | --- | --- |
| SC | UUCUCCGAACGUGUCACGUdTdT | ACGUGACACGUUCGGAGAAdTdT |
| Ifitm3 | GAACUACUGUGAUCAACAUdTdT | AUGUUGAUCACAGUAGUUCdTdT |
| β-Actin  Lamp2A  Hsc70  GAPDH | UGAAGAUCAAGAUCAUUGCdTdT  GCAGTGCAGATGAAGACAA  CTCCTCTTTCCCTTGGTATTG  CCCCTTCATTGACCTCAACTACA | GCAAUGAUCUUGAUCUUCAdTdT  GGCGCTTGAGACCAATAAA  GTAGGTGGTGAAAGTCTGTG  TCCCATTCTCAGCCTTGACTGT |

SC: Scramble control.

**2.2 Supplementary Table 2: Antibodies used in immunostaining and western blot**

| **Antibodies** |  | **Source** | **Dilution** |
| --- | --- | --- | --- |

| **Immunostaining** | |  | |  | |  |
| --- | --- | --- | --- | --- | --- | --- |
| IFITM3 | | Proteintech, 11714-1-AP | | 200 | |  |
| BCL2 | | Proteintech, 12789-1-AP | | 200 | |  |
| ATG7 | | Proteintech, 10088-2-AP | | 200 | |  |
| LAMP1 | | Proteintech, 21997-1-AP | | 200 | |  |
| LC3 A/B | | Proteintech, 66139-1-Ig | | 200 | |  |
| p62/SQSTM1 | | CST, #39749 | | 200 | |  |
| **Western blot** | |  | |  | |  |
| Cyclin D1 | | Proteintech, 60186-1-Ig | | 1000 | |  |
| Lamin B1 | | Proteintech, 12987-1-AP | | 5000 | |  |
| P70(S6K) | | Proteintech, 14485-1-AP | | 1000 | |  |
| Phospho-p70 S6 Kinase (Thr421/Ser424) | | CST, #9204 | | 1000 | |  |
| LAMP1 | | Proteintech, 21997-1-AP | | 1000 | |  |
| ATG13 | | Proteintech, 18258-1-AP | | 1000 | |  |
| c-MYC | | Proteintech, 10828-1-AP | | 1000 | |  |
| Beclin1 | | Proteintech, 11306-1-AP | | 1000 | |  |
| RHEB | | Proteintech, 15924-1-AP | | 1000 | |  |
| LC3 A/B | | Proteintech, 66139-1-Ig | | 1000 | |  |
| mTOR | | Proteintech, 20657-1-AP | | 500 | |  |
| p-mTOR (Ser2448) | | CST, #5536 | | 1000 | |  |
| AKT | | CST, #9272 | | 1000 | |  |
| p-AKT(Ser473) | | CST, #9271 | | 1000 | |  |
| p62/SQSTM1 | | CST, #39749 | | 1000 | |  |
| LAMP2 | | Proteintech, 66301-1-Ig | | 1000 | |  |
| P53 | | Proteintech, 10442-1-AP | | 2000 | |  |
| p44/42 MAPK(ERK1/2) | | CST, #4695 | | 1000 | |  |
| Phospho-p44/42 MAPK(ERK1/2) (Thr202/Tyr204) | | CST, #4370 | | 1000 | |  |
| Hsc70 | | Proteintech, 10654-1-AP | | 1000 | |  |
| Alexa Fluor 488/555 conjugated secondary antibodies | | Thermo Fisher Scientific,  A-10680; A-21422 | | 1000 | |  |
| HRP-conjugated β-Actin Antibody | | Proteintech, HRP-60008 | | 10000 | |  |
|  |  |  |  | |  | |

## 2.3 Supplementary Figures and Supplementary Figure Legends

**
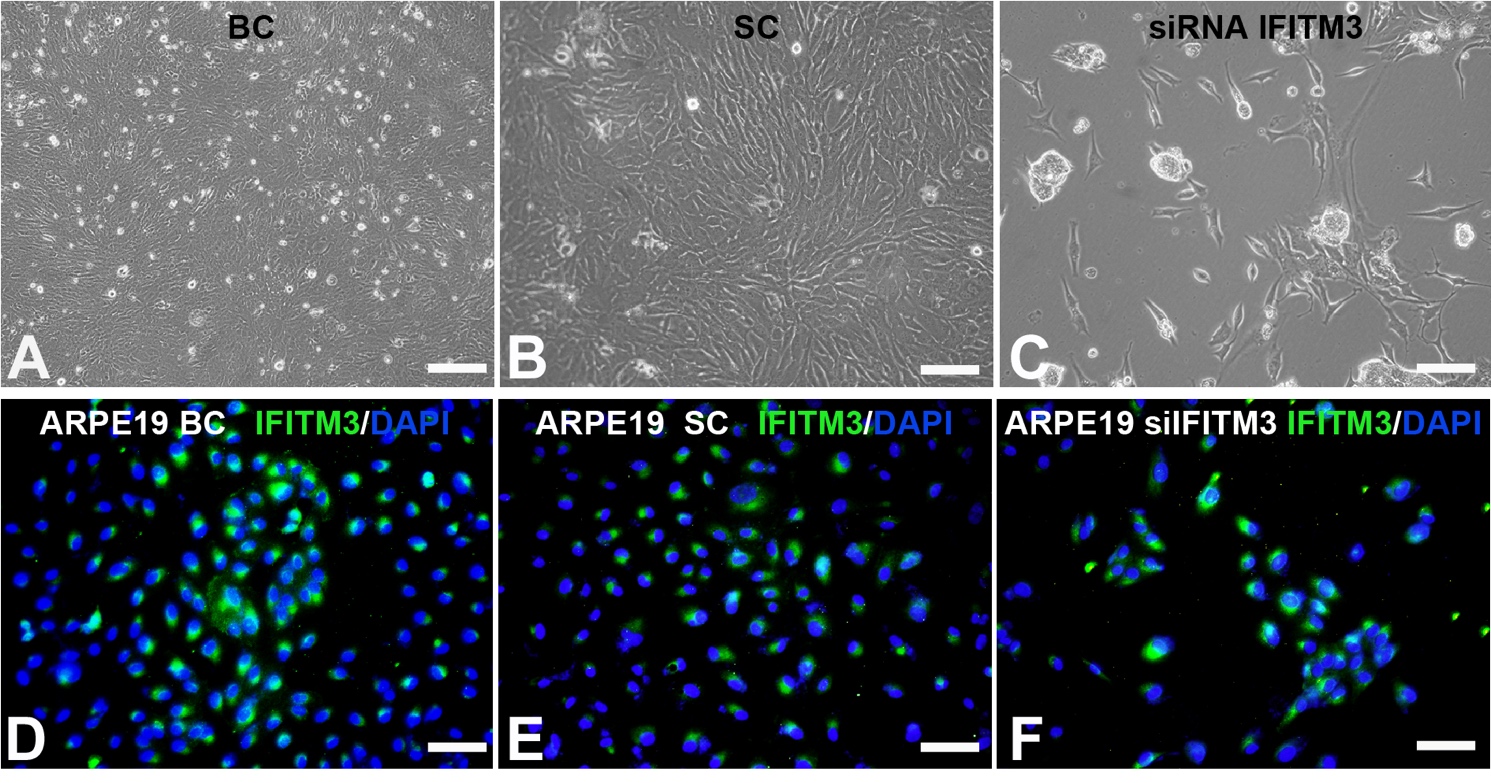
**

**Figure S1: Cell morphology and expression of IFITM3 of mNRPCs after knockdown with siRNA for 48h.**

**(A-C)** Cell morphology of the ARPE19 cells treated with siRNA transfection. (A) Black control group (BC), (B) Scramble control group (SC), (C) IFITM3-knockdown group (siRNA IFITM3). **(D-E)** Representative images of immunofluorescence of IFITM3 in ARPE19 cells. Magnification: ×200; scale bar: 100 μm.


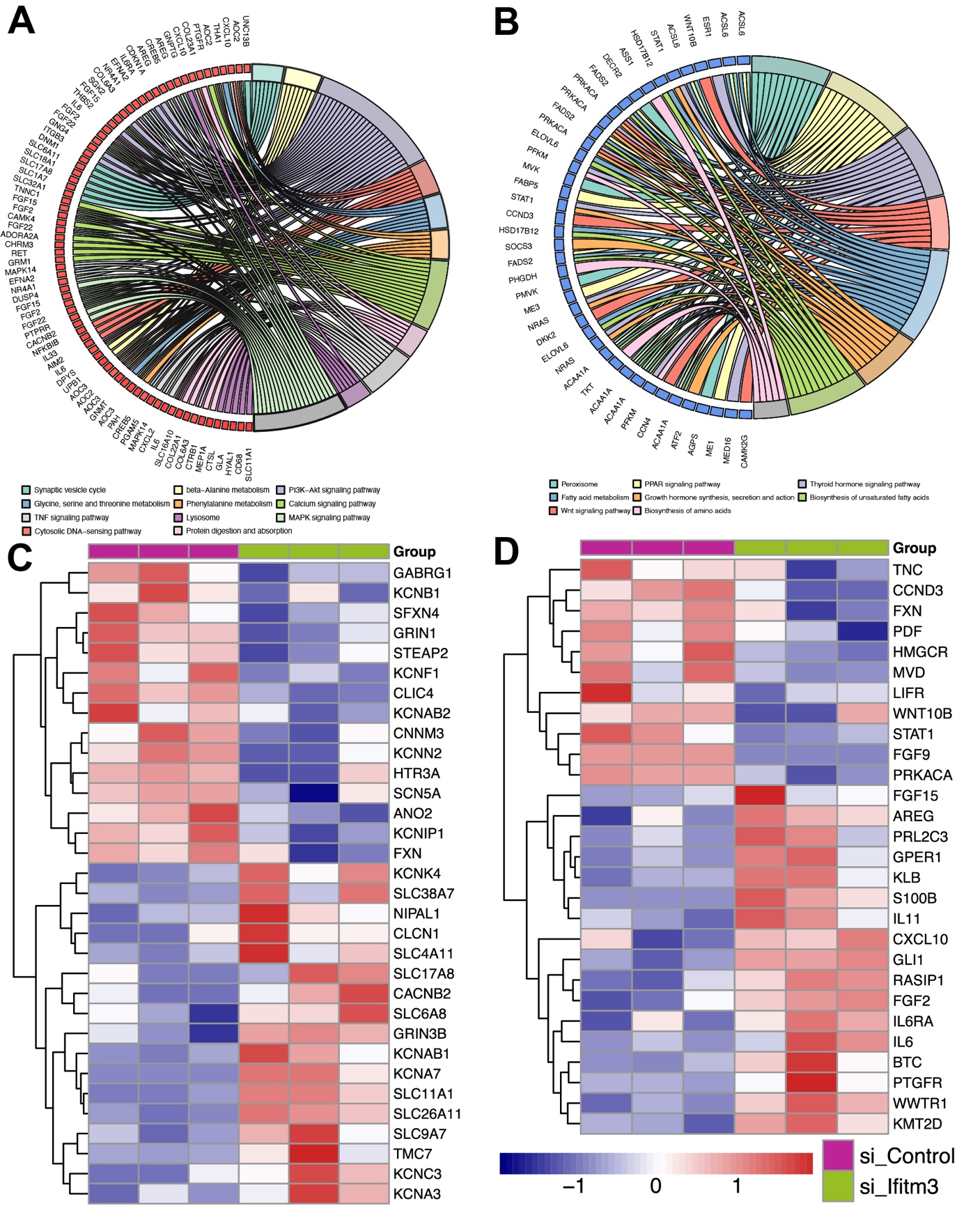


**Figure S2: High-throughput sequencing analysis of IFITM3-knockdown cells.**

**(A-B)** KEGG analysis showed the signaling pathways enriched in differentially expressed genes (DEGs). **(C-D)** Heatmap revealed the differentially expressed genes with related signaling pathways.

**
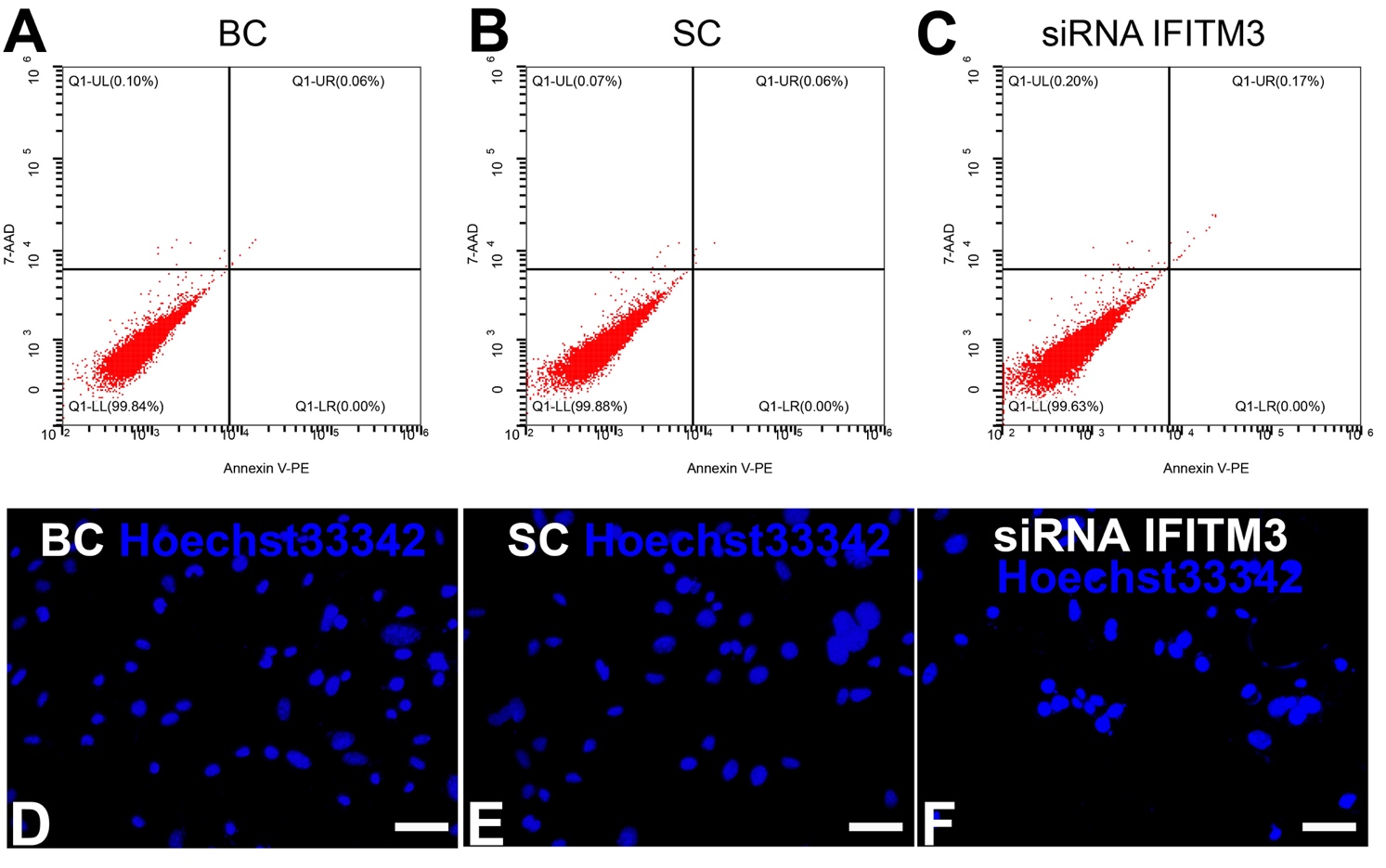
**

**Figure S3: Cell apoptosis assay after IFITM3-knockdown for 48h.**

**(A-C)** Flow cytometry assay of Annexin V-FITC staining in BC, SC and IFITM3-knockdown groups to determinate cell death. **(D-F)** Hoechst33342 staining in BC, SC and IFITM3-knockdown groups. Magnification: × 200; scale bar: 100 μm.


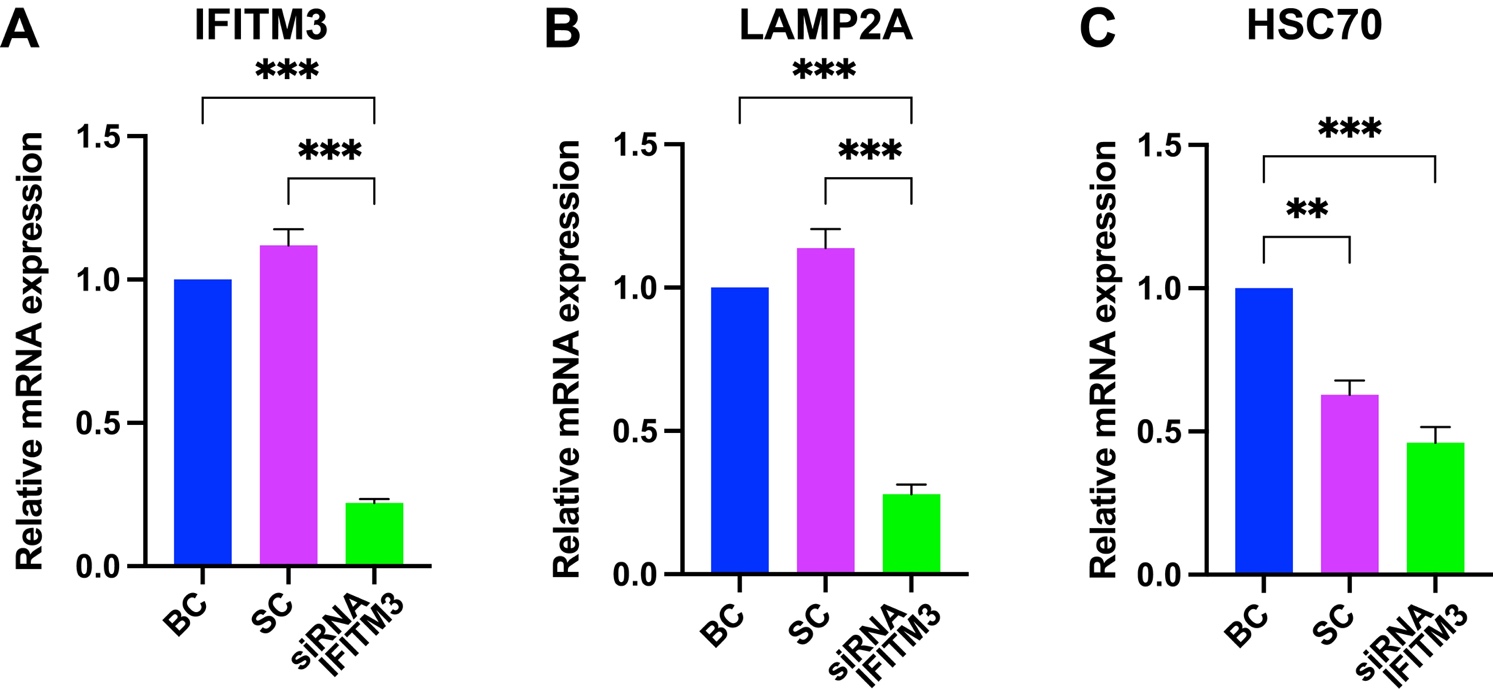


**Figure S4: qRT–PCR assay of LAMP2A and HSC70 expression in mNRPCs after knockdown IFITM3 for 48h. (A-C)** qRT–PCR assay results of LAMP2A, HSC70 and IFITM3 in BC, SC and IFITM3-knockdown groups. Data are presented as the mean ± SD (n = 3). *: P < 0.05; **: P < 0.01 (one-way ANOVA and Sidak’s multiple comparisons test).

**
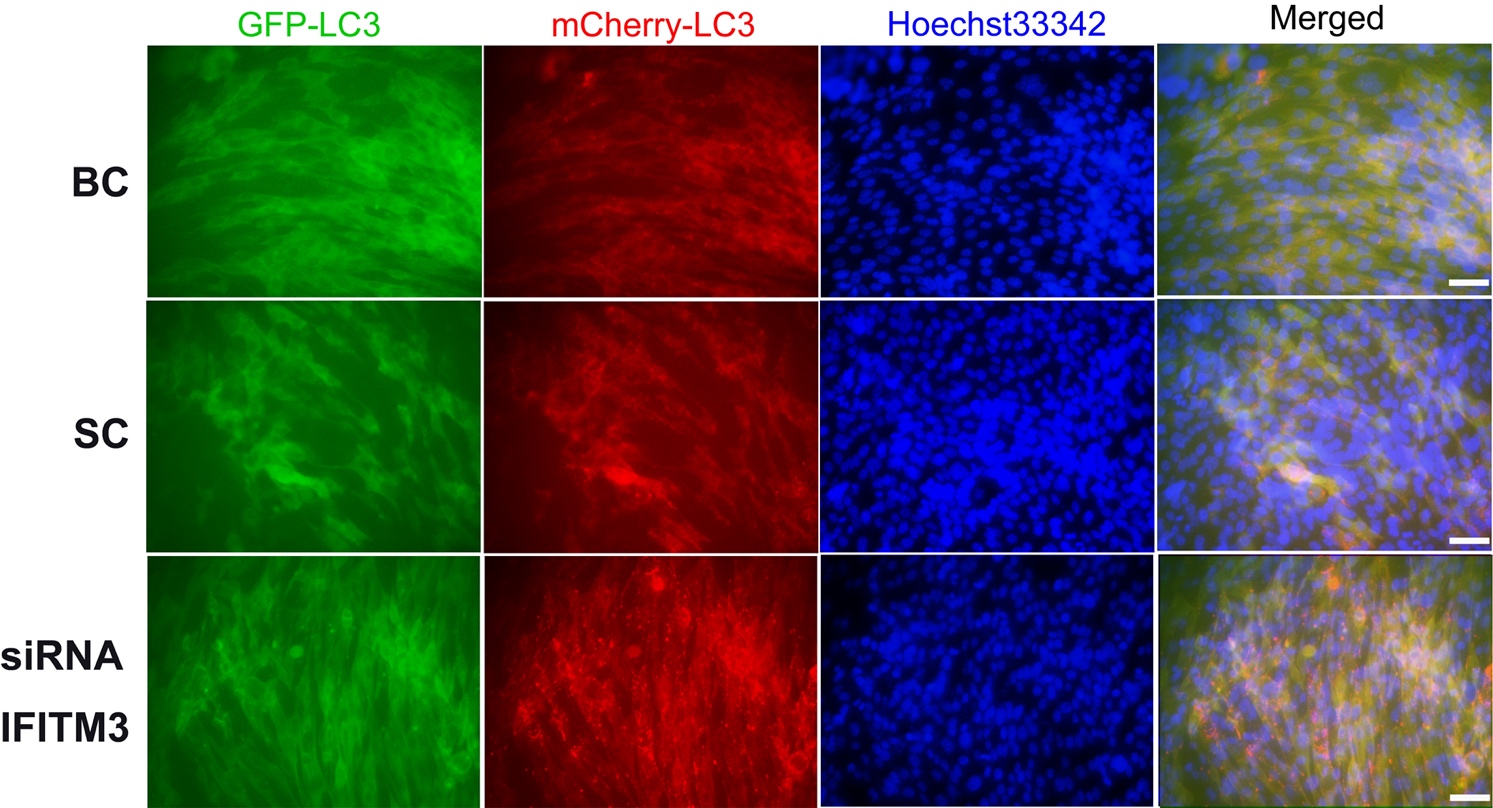
**

**Figure S5: Fluorescent images showing mCherry-GFP-LC3 expression after IFITM3 knockdown used to assay autophagic flux in the cells.**
